# Analyzing Joshimath’s sinking: causes, consequences, and future prospects with remote sensing techniques

**DOI:** 10.1038/s41598-024-60276-3

**Published:** 2024-05-13

**Authors:** Shubham Awasthi, Kamal Jain, Sashikanta Sahoo, Rohit Kumar, Ajanta Goswami, Girish Chandra Joshi, Anil V. Kulkarni, D. C. Srivastava

**Affiliations:** 1 Centre of Excellence in Disaster Mitigation and Management, Indian Institute of Technology Roorkee, Roorkee, Uttarakhand India; 2 Civil Engineering Department, Indian Institute of Technology Roorkee, Roorkee, Uttarakhand India; 3 Department of Earth Sciences, Indian Institute of Technology Roorkee, Roorkee, Uttarakhand India; 4 Uttarakhand State Disaster Management Authority, Dehradun, Uttarakhand India; 5 Divecha Centre for Climate Change, Indian Institute of Science, Bengaluru, Karnataka 560012 India

**Keywords:** Land deformation, Sinkage, Joshimath, Himalaya, Remote sensing, Environmental impact, Climate and Earth system modelling, Natural hazards

## Abstract

The Himalayas are highly susceptible to various natural disasters, such as the tectonically induced land deformation, earthquakes, landslides, and extreme climatic events. Recently, the Joshimath town witnessed a significantly large land subsidence activity. The phenomenon resulted in the development of large cracks in roads and in over 868 civil structures, posing a significant risk to inhabitants and infrastructure of the area. This study uses a time-series synthetic aperture radar (SAR) interferometry-based PSInSAR approach to monitor land deformation utilizing multi-temporal Sentinel-1 datasets. The line of sight (LOS) land deformation velocity for the Joshimath region, calculated for the year 2022–2023 using a PSInSAR-based approach, varies from − 89.326 to + 94.46 mm/year. The + ve sign indicates the LOS velocity/displacement away from the SAR sensor, whereas − ve sign signifies the earth's movement towards the SAR sensor in the direction of LOS. In addition, the study investigates feature tracking land displacement analysis using multi-temporal high-resolution Planet datasets. The result of this analysis is consistent with the PSInSAR results. The study also estimated the land deformation for the periods 2016–2017, 2018–2019, and 2020–2021 separately. Our results show that the Joshimath region experienced the highest land deformation during the year 2022–2023. During this period, the maximum land subsidence was observed in the north-western part of the town. The maximum LOS land deformation velocity + 60.45 mm/year to + 94.46 mm/year (2022–2023), occurred around Singhdwar, whereas the north and central region of the Joshimath town experienced moderate to high subsidence of the order of + 10.45 mm/year to + 60.45 mm/year (2022–2023), whereas the south-west part experienced an expansion of the order of 84.65 mm/year to − 13.13 mm/year (2022–2023). Towards the south-east, the town experienced rapid land subsidence, − 13.13 mm/year to − 5 mm/year (2022–2023). The study analyzes the causative factors of the observed land deformation in the region. Furthermore, this work assesses the ground conditions of the Joshimath region using UAV datasets acquired in the most critically affected areas such as Singhdhaar, Hotel Mountain View, Malhari Hotel, and Manoharbagh. Finally, the study provides recommendations and future prospects for the development policies that need to be adopted in the critical Himalayan regions susceptible to land deformation. The study suggests that land deformation in the region is primarily attributed to uncontrolled anthropogenic activities, infrastructural development, along with inadequate drainage systems.

## Introduction

The Himalayan mountain range, a consequence of the collision between the Indian and the Eurasian tectonic plates, is a tectonically and seismically active belt^1,2^. Here, land deformation is inevitable due to the forces exerted on the ground. The frequent earthquake occurrences further aggravate the land deformation process^3,4^. The Himalayas are also susceptible to landslides that are commonly triggered by earthquakes, heavy rainfall, and anthropogenic activities, such as deforestation and large-scale civil constructions^5,6^. The development of large infrastructural projects contributes to deforestation and slope destabilization, increasing the likelihood of landslides. Also, the continuous construction activities can lead to soil compaction and other changes in the ground, ultimately resulting in land deformation^7,8^. In summary, the land deformation in the Himalayas can be attributed to various factors, such as tectonic activity, earthquakes, landslides, deforestation, civil construction, and large infrastructural projects^9,10^.

Land subsidence, emergence, and sinkage can significantly impact the local environment and communities, leading to infrastructure damage, disruption in transport and communication networks and other vital services like water and electricity, and even loss of life in some cases. Recent land subsidence in the Joshimath area of the Higher Himalayas has resulted in the collapse of the individual as well as public infrastructures and loss of lives, leading to tremendous social insecurity among the local communities. According to reports published in various secondary sources, over 868 houses have developed cracks, and many houses have partially or completely collapsed^11^. People have to evacuate their homes and seek safer places of accommodation. The risk of house collapse may be amplified due to the severe winter and sudden landslide activity due to the unstable land surface. The cracks in the walls and floors of houses in the urban area are deepening with every passing day, creating an alarming situation for the inhabitants as well as authorities^12^. The disastrous event and its consequences call for an immediate quantitative assessment of the observed land subsidence in the area. For effective disaster risk management and sustainable development in the Himalayas, it becomes necessary to understand the causes of land deformation.

There are various ground observation and remote sensing-based methods for measuring land deformation, e.g., GPS surveys, LIDAR (Light Detection and Ranging) surveys, ground-based sensors, and satellite remote sensing techniques such as MT-InSAR (multi-temporal synthetic aperture radar interferometry)^13^ and Cossi-Corr (Coregistration of optically sensed images and correlation)^14^ based methods. The MT-InSAR approach uses time-series Synthetic Aperture Radar datasets, whereas the Cossi-Corr method uses multi-temporal optical imageries to measure surface dynamics. GPS technique utilizes satellite signals to accurately measure the position of points on the ground, and the measurement of their positions over time detects any movement or deformation of the ground^15^. LIDAR technique uses lasers to measure the distance between the LiDAR sensor and the ground.

A high-resolution 3D map of the ground surface can be generated by measuring the distance from the datum to the ground at numerous points over a given area, and small changes in the ground could be detected by comparing these maps over time. Further, many types of ground-based sensors, such as inclinometers, extensometers, and piezometers, can be employed to gauge land deformation^16^. These sensors can measure small movements or changes in the ground surface and monitor land deformation over time. However, ground-based deformation measurement using GPS surveying and LIDAR based methods is very challenging for continuous monitoring due to the highly rugged terrain and extreme weather conditions in the higher Himalayas^17,18^. During most of the year, these locations are inaccessible due to high snow cover^19^. The GPS sensor network has continuous data procurement, connectivity, and consistency issues. However, satellite remote sensing offers a cost-effective technique that obtains long-term data for monitoring dynamic changes without requiring significant manpower^20,21^. Therefore, it acts as an efficient tool for continuously monitoring land deformation processes^22^.

Various studies have also used Cossi-Corr based methods for measuring surface dynamics happening due to geological processes, climate change scenarios, or anthropogenic activities^14,23–25^. Time-Series InSAR-based methods, like PSInSAR, provide high-resolution land deformation measurements with very high accuracy, nearly on a millimeter scale^26–28^. The PSInSAR method was initially developed by Ferretti et al. in 2007 based on the approach of selecting permanent scattering candidate information from the interferometric stack using amplitude dispersion methods^29^. Further, Hooper et al.^30^ enlarged the scope of the PSInSAR method by including spectral phase diversity data in the Stanford Method for Persistent Scatterers (StaMPS)^31^. These improvements prompted Tamburini et al. to use PSInSAR technique for monitoring and comprehending the dynamic behavior of subsurface reservoirs in terms of volumetric changes^31^. They compared the PSInSAR results with traditional surveying techniques and concluded that the PSInSAR method was providing a higher number of measurement points per square km with good accuracy and was having low costs over long periods.

The PSInSAR has been used to analyze earth surface dynamics and processes including land subsidence, groundwater level change, earthquakes, and landslides^28,32,33^. Subsequently, another study used PSInSAR for analyzing the extremely long-term spatiotemporal evolution of land subsidence in the region of Taiyuan, China, and concluded that ground deformation was concentrical around the areas of high groundwater loss^34^. Later, PSInSAR and geographic spatial analysis were used to identify and describe subsidence changes in the Beijing Plain^35^. The equal fan analysis method (EFAM) revealed that the maximum expansion occurred in an eastward direction of the Beijing Plain^35^. The regions with the highest subsidence correlated with the areas where groundwater levels were declining in the Beijing Plain^35^. Another study also tried to estimate land deformation induced due to groundwater withdrawals and examined the patterns between groundwater extraction and its associated land subsidence in the Qazvin plain of Iran using ENVISAT and Sentinel-1 time-series datasets applying PSInSAR technique^36^.

Razi et al.^37^ employed PSInSAR method for extracting earthquake-induced land deformation in Chiba prefecture utilizing time-series ALOS-2 PALSAR-2 SAR datasets. Later, Kumar et al.^38^ investigated urban damage and terrain deformation caused by the 2015 earthquake that struck Kathmandu, Nepal used PSInSAR with Sentinel-1 SAR datasets. In addition, Liu et al.^39^ recovered the temporal and geographical variations of ground settlement associated with land reclamation due to the soil consolidation and water recharge at the Xiamen New Airport in China using C-band Sentinel-1 satellite datasets. In another study, Crustal Deformation of the Weihe Basin during 2015–2019 was estimated by applying PSInSAR with C-band Sentinel-1A/B and L-band ALOS PALSAR-2 ScanSAR imagery. Taloor et al.^40^ analyzed the rate of crustal deformation in the Kashmir basin using Sentinel-1 SAR datasets employing PSInSAR approach. Their analysis showed the dominant uplift in the valley and surrounding hill ranges^40^. Awasthi^41^ used the PSInSAR method to measure land deformation caused by groundwater stress as a result of fast urbanization. This study successfully differentiated the specific areas experiencing land subsidence using ground water and surface water as sources based on observed urban land subsidence in the region. All these studies showed good potential of PSInSAR technique in quantifying land deformation induced due to landslides, earthquakes, groundwater withdrawals etc. using time-series multi-temporal InSAR datasets.

With the advancement in remote sensing techniques, the optical satellite data has been found useful in capturing the movement of ground motion with some limitations^42–44^. Optical remote sensing has also shown its potential in monitoring ground motion through feature tracking approaches^45,46^. The Cossi-Corr method, a displacement measurement technique based on feature tracking, has been frequently employed for accurately measuring surface displacement associated with geomorphological and tectonic processes at a sub-pixel level of precision utilizing multi-temporal optical satellite datasets^25,47–49^. Various studies have used Cosi-Corr method for mapping displacement induced due to landslides^49–54^. Also, Cosi-Corr methods showed a great ability in demonstrating the slow-motion mass movement^51^. Despite their comprehensive utility, the optical remote sensing-based techniques have some significant limitations, e.g., orthorectification, co-registration error, High-Hill shadows, very dense vegetation coverage, Cloud cover, very high movements, and unavailability of data^55^.

This study aims to provide new insights into the causes of the recent land deformation process in Joshimath town. This study used PSInSAR and Cosi-Corr approaches on time-series Sentinel-1 C-band datasets and multi-temporal Planet scope optical satellite images for the quantitative assessments of land deformation. The results are interpreted in the backdrop of the geological setting of the Joshimath region. The study provides possible causes of the occurring land deformation like the anthropogenic activities, urbanization, and infrastructure growth that have occurred in the region in recent years, along with the UAV based analysis of the cracks sites.

## Study area details and dataset used

**Figure 1:**
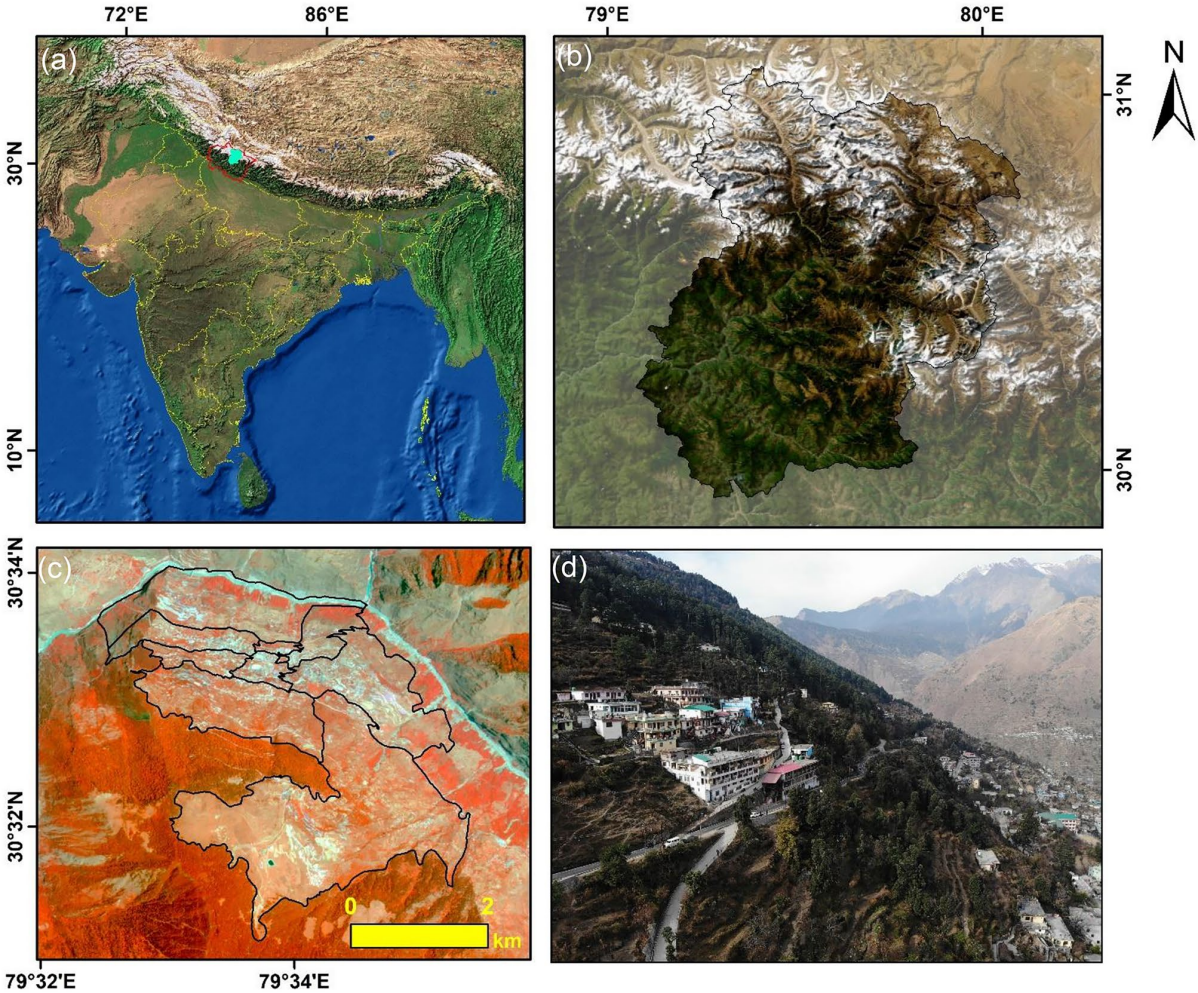
Study region: (**a**) Location of the Chamoli District in Uttarakhand state in India; (**b**) Chamoli District on Google Earth Image; (**c**) Present Municipal Boundary and Wards of Joshimath Town; (**d**) Aerial View of Joshimath (This figure is generated using ArcGIS software-version 10.3.1https://enterprise.arcgis.com/en/portal/10.3/use/deploy-app-portal-obsolete.htm).

The study illustrates the landscape of the Joshimath area using Google Earth images, Planet-scope dataset and UAV acquired aerial imageries (Fig. 1). The study area, in and around Joshimath town (79.56° N; 30.55° E), is situated in the Higher Himalaya in the Chamoli District of Uttarakhand state, India The town is located on the north-west bank of the river Alaknanda ^56^, a major tributary of river Ganges. The main town of Joshimath is situated between the altitude ranges of 1500–2100 m, on the north-facing slope, with a gradient varying between 30° and 40°. The municipal boundaries of Joshimath are the vegetated high mountain ridges in the north and is flanked towards the heights of Auli (2800 m) in the south. The region has tremendous geographic, ecological as well as religious significance attracting a lot of tourists for a major part of the year. The town has a population of approximately 22,900 people and it caters to large number of pilgrims and tourists throughout the year. It can be reached by National Highway No. 58. However, in the past few months, Joshimath has gained a new and alarming situation among its 22,900 residents as a town that may still be sinking^57^.

## Study area

### Geology, geomorphology and lithology

**Figure 2:**
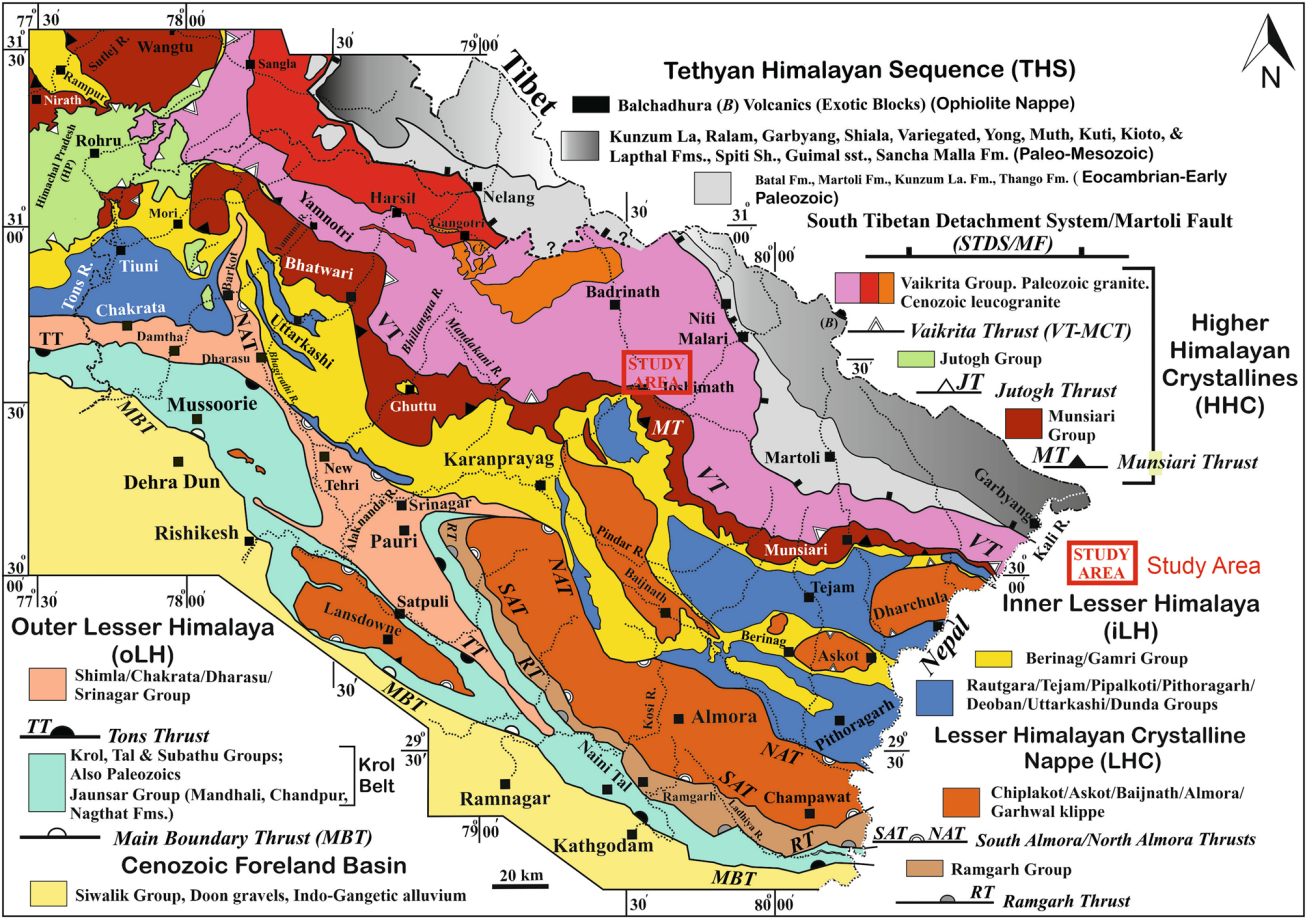
Geology map of the study region^61^ (This figure is generated using CorelDraw Graphics Suite 2019; https://www.coreldraw.com/).

The Joshimath lies in the most vulnerable seismic Zone-V in the Himalaya^58^. The town is built upon approximately 0.5 thick debris paleo-landslides. The bed rocks are highly sheared, repeatedly folded, fractured, jointed, and faulted gneisses of the Higher Himalaya. Notably, the town lies in the vicinity of two intracontinental thrusts, the Munsiari Thrust and the Main Central Thrust/Vaikrita Thrust (Table 1). The Munsiari Thrust emplaces a variety of Lesser Himalayan gneisses and schists over the Lesser Himalayan metasedimentary rocks^59–61^ (Fig. 2). The Main Central Thrust runs along the interface of two different gneisses, ~ 1.95–1.89 Ga and 0.06–0.86 Ga, that belong to the Munsiari Group and the overlying Vaikrita Group (Table 1). The town Joshimath is situated upon the debris that overlie the Joshimath Formaton of the Vaikrita Group^61^. The town of Joshimath lies on highly water-saturated paleo-landslide debris, which in turn overlies highly fractured and sheared schist, gneiss, and quartzite^62,63^.

**Table 1:** Lithological units details.

Lithology around Joshimath		
Vaikrita group	Bhapkund/Badrinath Fm	High grade gneiss, and amphibolite
	Suraithota/Pandukeshwar Fm	
	Joshimath Fm	
Main Central Thrust/Vaikrita Thrust		
Munsiari group	Munsiari group	Low to medium grade augen gneiss, schist, quartzite and amphibolite
Munsiari Thrust (MT)		
Berinag formation	Berinag Fm	Quartzite with interbanded amphbolite

**Figure 3:**
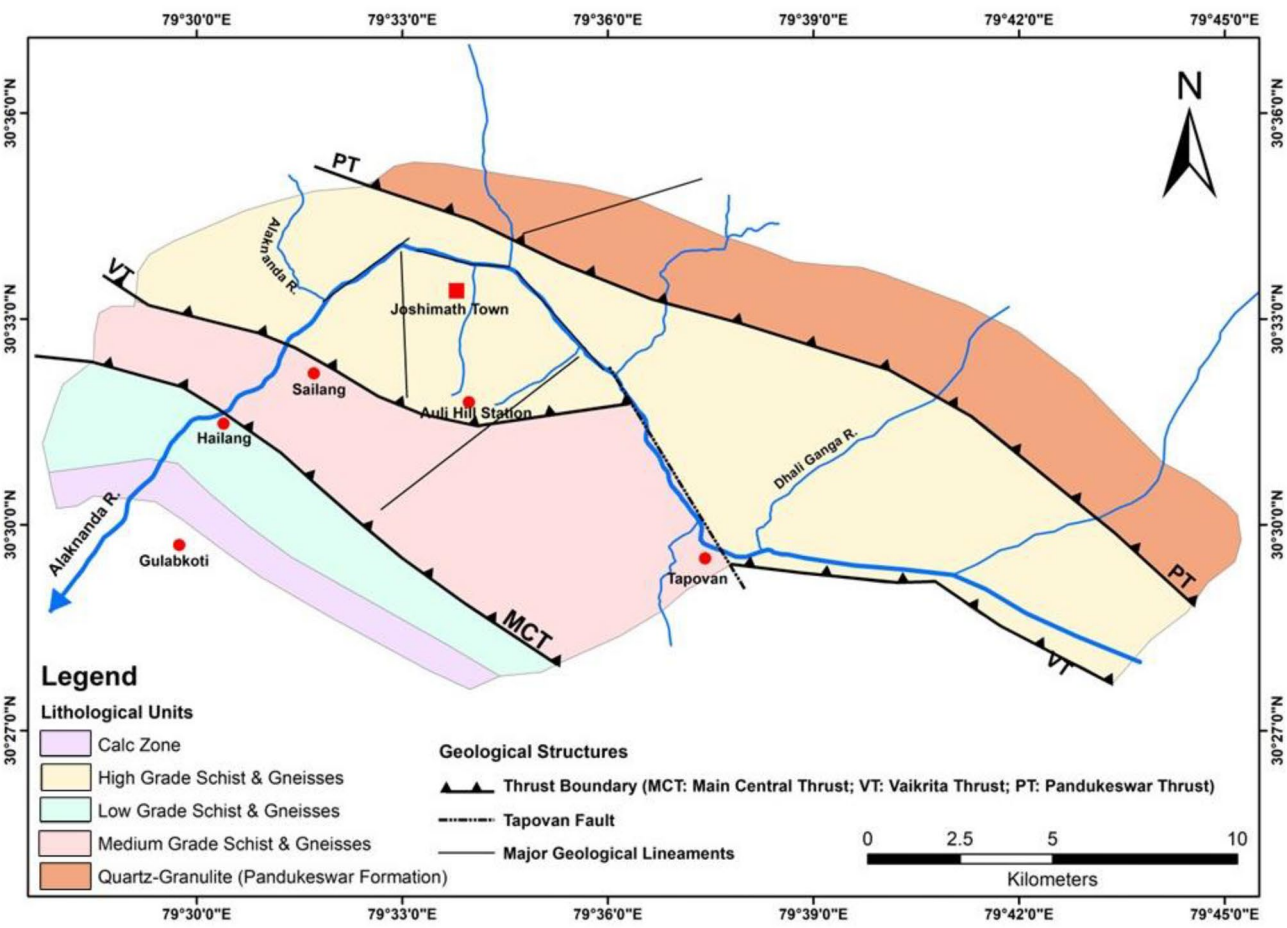
Detailed Geology Map (Lithological units and Geological structures) of Joshimath Region (Modified and recreated after Valdiya^64^) (This figure is generated using ArcGIS software-version 10.3.1 https://enterprise.arcgis.com/en/portal/10.3/use/deploy-app-portal-obsolete.htm).

The paleo-landslide deposits, combined with unstable slope deposits, are susceptible to future failure in the Joshimath region. Although the movement of old mass and paleo-landslide deposits is very slow, it may be triggered by small factors like overburden on a slope, interference due to major infrastructure development, seismicity, and rainfall^62,63^. The geological and geophysical setting of Joshimath is not favourable for construction and requires scientific methods for limited ecology/terrain controlled construction and stabilization. The region is characterized by a series of ridges, with heights decreasing progressively comprising both the Greater and Lesser Himalaya, and featuring limited-level land. The geology map of Joshimath Region, is shown in Fig. 3. The soil in the region is natural, dynamic, and heterogeneous. It supports plant, agriculture, and ecosystem diversity. These soils have developed from various types of rocks, such as granite, schist, gneiss, phyllites, shales, and slate, under cool and moist conditions. The steep to very steep hills slopes and Glacio-fluvial valleys are primarily composed of shallow to moderately shallow and excessively drained soils that are sandy-skeletal to loamy-skeletal in nature, with a neutral to slightly acidic pH and low water retention. Classified as Lithic/Typic Cry orthents, these soils are generally found in areas with sparse vegetation. Table 1 shows the lithological units in the region and their respective metamorphic grades^61^.

### Local topography

**Figure 4:**
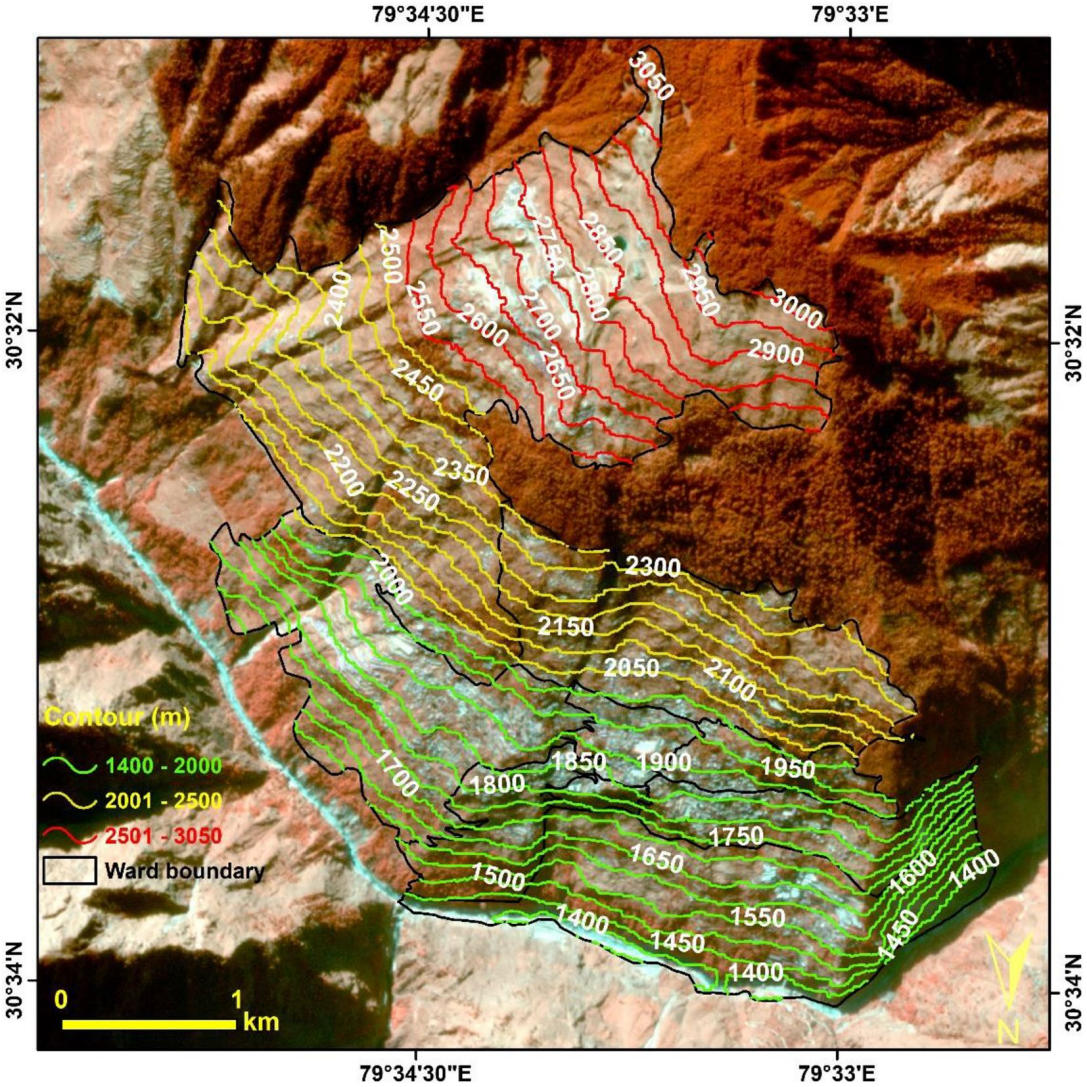
The Contour map showing the elevation changes in the Joshimath area. (This figure is generated using ArcGIS software-version 10.3.1https://enterprise.arcgis.com/en/portal/10.3/use/deploy-app-portal-obsolete.htm).

The elevation in the Joshimath Municipal boundary region varies between 1400 to 3050 m, extending from the Alaknanda river in the valley all the way up to Auli peaks, as shown in Fig. 4 that is prepared using the Planetscope background image and CartoDEM V3R1 (30 m). The major habilitated region in the town is between the altitude of 1700–2000 m. The area in this zone is very densely populated, and most of the Joshimath town houses construction is done in this region. Further, contour lines show a very high elevation change that has resulted in slope instability in the region.

### Physiography and climate

The physiography of the region comprises high hills and mountains along with the Alaknanda river catchment in the Higher Himalaya. The topography of the Joshimath region consists of highly elevated, rugged, mountainous terrain that comprises deep valleys and steep cliffs with an average elevation of more than 6100 m. The climate in the region varies from sub-tropical monsoon type in some areas to tropical upland type in others^65–67^. The northern part of the region is characterized by severe winters and higher rainfall^68^. The year can be divided into four distinctive seasons: the winter season, which is characterized by cold weather from December to February, the warm season from March to May, the monsoon season brought by south-west winds from June to September, and the post-monsoon season from October to November. The maximum and minimum temperatures range from − 2.9 to 37 °C^69^. The town of Joshimath receives an average precipitation of 76.88 mm annually, with 132.27 rainy days, which is 36.24% of the total year (Source: IMD)^7,70^.

### Satellite datasets

#### Sentinel-1 SAR datasets

**Figure 5:**
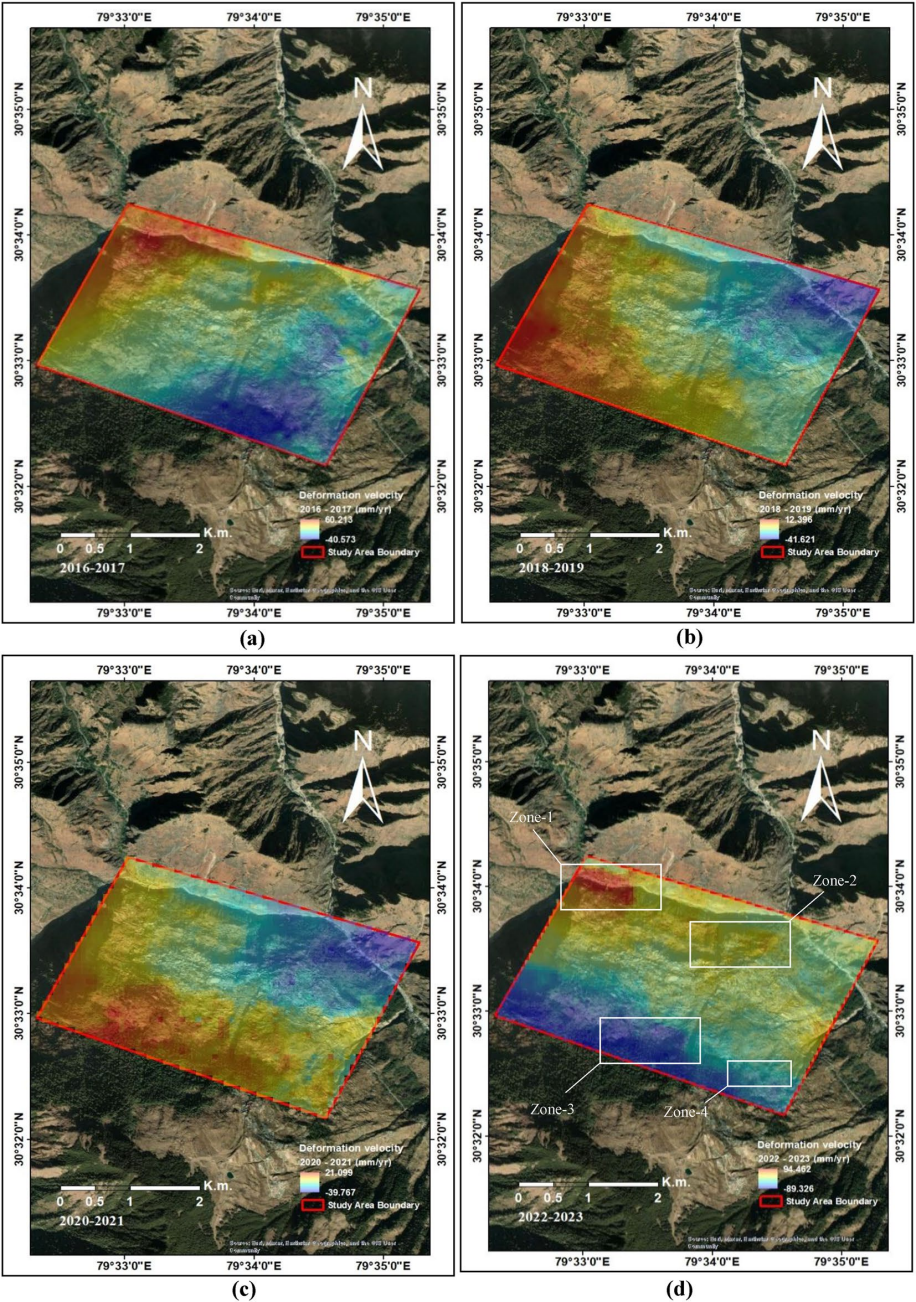
(**a**) Estimated land deformation in the Joshimath region during 2016–2017, (**b**) Estimated land deformation in the Joshimath region during 2018–2020, (**c**) Estimated land deformation in the Joshimath region during 2021–2022 (**c**) Estimated land deformation in the Joshimath region during 2022–2023 (This figure is generated using ArcGIS software-version 10.3.1https://enterprise.arcgis.com/en/portal/10.3/use/deploy-app-portal-obsolete.htm).

Sentinel-1 is a radar imaging satellite mission of the European Space agency (ESA). Sentinel-1 Synthetic Aperture Radar (SAR) satellite datasets, used in this study, have a global repeativity of 12 days^71^. These radar remote sensing-based active sensors have the advantage of all-weather earth observations, even during cloud cover, snowfall, and hazy weather conditions^72^. Figure 5 shows the spatial distribution and temporal distribution of the used datasets using the Normal baseline and the temporal baseline information of the time-series SAR datasets. For quantitative assessment and estimation, total 83 Sentinel-1 datasets between 12 May 2016 and 17 September 2023, covering the abnormal and abrupt land subsidence phenomenon in the study region. Based on the information gathered from field surveys and a ground survey of the study site, the region is reported to be experiencing the highest and most frequent cases of land deformation, creating an alarming situation during this period. For estimating the recent land subsidence activity 29 datasets from the years 2022–2023 were used. For the comparative analysis of the observed land subsidence in the region, 12 SAR datasets forming the interferometric stack for the year 2016–2017, 24 SAR interferometric stack datasets for the years 2018–2019 and 16 time-series SAR datasets of the year 2020–2021 are used for further comparing them with the estimated land deformation for the 2022–2023 period.

#### Optical datasets and digital elevation models (DEM) datasets

Optical datasets and DEM are commonly used for land subsidence studies^73^. Optical imagery, such as satellite or aerial photographs, can identify visible signs of subsidence, such as cracks or changes in surface features. DEM provides a quantitative representation of the topography of a region, which can be used to track changes in elevation over time and detect subsidence. By combining optical imagery and DEM, one can accurately identify and quantify subsidence, which can be caused by factors such as groundwater extraction, oil and gas extraction, or natural processes. Understanding the extent and causes of subsidence is critical for managing and mitigating its impacts on infrastructure, natural resources, and communities. Table 2 gives details of the Optical and DEM datasets used in the study.

**Table 2:** Optical and DEM dataset details.

Datasets	Acquisition date	Spatial resolution (m)	Purpose
CartoDEM V3 R1	2012	1 arc-second (30 m)	Slope, contour and aspect estimation drainage extraction
Landsat-8 datasets	23 November 2022 25 December 2022 10 January 2023	30 m	Displacement calculation using Cosi-Corr method
Planet datasets	04 December 2020 04 December 2021 04 June 2022 28 December 2022 07 January 2023	3 m	Mapping of building Footprint Displacement estimation using Cosi-Corr using different year combination
Declassified CORONA Datasets KH-4B	24 November 1970 08 September 1980	1.8 m	Urban area and other feature identifications and mapping purpose

## Methodology

### Time-series InSAR based persistent scatterer interferometry SAR (PSInSAR) processing

During the estimation of land deformation velocity, the Sentinel-1 datasets were pre-processed by analyzing the Sentinel-1 SLC SAR datasets based on spatial baselines (B_⊥_) and temporal baselines (B_Temp_), selecting a master image, co-registering all the Sentinel-1 images with the master image, and applying a DEM assisted geometric correction. Next, the interferogram formation was carried out by calculating the phase difference between two complex SAR observations^41^. Then, the Sentinel-1 TOPS data was combined, and individual bursts present in the datasets were merged. Furthermore, the topographic phase was removed using the SRTM (3 arc-seconds) DEM. Finally, the processed time-series SLC datasets were transformed and exported into binary raster StaMPS format files for further land deformation estimation using StaMPS^22^.

The StaMPS method was applied to calculate the land deformation velocity by selecting Persistent Scattering (PS) pixels. These are the pixels with a persistent high interferometric complex correlation in a time-series interferometric stack^74^. The procedure consisted of four stages: creating interferograms, determining phase stability, identifying a network of stable pixels which are self-consistent in the interferometric stack, and estimating the land deformation velocity along the line of sight^74^. The overall composite interferometric coherence is described as follows^30^:

1 $$\rho_{total} = { }\rho_{{\text{temporal }}} {* }\rho_{{\text{spatial }}} {* }\rho_{{\text{Doppler }}} {* }\rho_{{\text{thermal }}}$$

where $\rho_{{\text{temporal }}}$ is temporal correlation, $\rho_{{\text{spatial }}}$ is spatial correlation, $\rho_{{{\text{doppler}} }}$ denotes the doppler frequency correlation, and $\rho_{{\text{thermal }}}$ represents the thermal correlation.

The selection of PS pixels was carried out using an amplitude dispersion index and a phase-based approach. The amplitude dispersion index $\left( {D_{A} } \right)$ is calculated as the ratio of the standard deviation ($\sigma_{A}$) to the mean $\left( {\mu_{A} } \right)$ of the amplitude values for each pixel in the set of SLC images. The phase-based approach considers the components that make up the interferogram, including the deformation phase, phase due to precise orbit errors, atmospheric phase screen, topographic correction errors, and uncorrelated noise-phases^28^. The deformation phases exhibit minimal spatial and temporal correlation. In contrast, atmospheric phase screens and precise orbit phases have low spatial correlation but high temporal correlation. Additionally, topo-phase correction errors and uncorrelated noise phases display high spatial correlation and low temporal correlation. Lastly, uncorrelated noise phases exhibit high spatial correlation and low temporal correlation^30,75^. The following equations are used to estimate the PS candidates in the InSAR stack^30,41^:

2 $$D_{A} = \frac{{\sigma_{A} }}{{\mu_{A} }}$$

3 $$\varphi_{x,i} = w\left\{ {\varphi_{D,x,i} + { }\varphi_{A,x,i} + { }\Delta \varphi_{S,x,i} { } + { }\Delta \varphi_{{{\uptheta },x,i}} { } + { }\varphi_{N,x,i} { }} \right\}_{{2{\uppi }}}$$

Here, the $\varphi_{x,i}$ represents the wrapped phase related to *x*th pixel in the *i*th interferogram; $\varphi_{D,x,i}$ denotes the Phase variation because of pixel displacement in the direction of flight; $\varphi_{A,x,i}$ is the phase due to the atmospheric delay; $\Delta \varphi_{S,x,i}$ signifies residual phase caused by errors in the satellite orbit and the external DEM; $\Delta \varphi_{{\uptheta ,x,i}}$ is the consequent phase due to the inaccurate look angle; $\varphi_{N,x,i}$ denotes the phase noise created in a SAR resolution cell because of uncorrelated non-dominant scatterers; **w** denotes the wrapping factor, which ascertains that the phase value wrapped with the 2π^30,76^. The residual uncorrelated look angle error is reduced by eliminating the topographic phase error. Furthermore, the residual phase's stability ($\Upsilon_{{\text{X}}} )$ was assessed as follows:

4 $${\Upsilon }_{{\text{X}}} = \frac{1}{{\text{N}}}\left| {\mathop \sum \limits_{i = 1}^{N} exp\left\{ {\sqrt { - 1} \left( {\varphi_{x,i} - \tilde{\varphi }_{x,i} - { }\Delta \varphi_{{\theta_{x,i} { }}}^{u} } \right)} \right\}} \right|$$

Here, *N* quantifies the total number of interferograms, $\tilde{\varphi }_{x,i}$ represents the wrapped estimation of spatially correlated elements of the interferometric phase $\varphi_{x,i}$, signifies the satellite inaccuracy phase error, atmospheric error phase, and the phase resulting due to the look angle error. The term $\Delta \varphi_{{\theta_{x,i} }}^{u}$ is defined as the approximate unwrapped topographic phase error^30,76^. Additionally, the residual topographic phase error, caused by removing the spatially uncorrelated look angle error or DEM error from the phase of the chosen PS pixels, is eliminated^30,76^.

The spatio-temporal filters were employed to isolate each phase component. After the phase unwrapping process, the spatial correlation of the look angle error (SCLA) in the persistent scattering pixels is calculated. Applying high-pass time and low-pass space filters on the unwrapped phase data yields the SCLA error^41^. The residual phase resulting from deformation is created by subtracting the SCLA error from the still present phase. Hence, using the StaMPS approach, a one-dimensional line of sight (1D-LOS) velocity vector is determined for the area of study^22,30^.

### COSI-Corr method for measuring ground deformation using optical satellite datasets

The co-registration of Optically Sensed Images and Correlation (COSI-Corr) method is a remote sensing technique commonly used to determine land subsidence, tectonic displacement, glacier velocity, dune migration, and landslide movements^48,51^. The method uses high-resolution optical satellite images to identify changes in the landscape over time, such as the movement of slow-moving landslides and the advancement of glaciers^53^. The Cosi-Corr method is based on the principle of cross-correlating two or more images of the same area taken at different times to identify displacement areas. Analyzing these displacements can provide insight into the processes driving the mass movements in the study area^54^.

The COSI-Corr method is a sub-pixel-matching method, and the main correlation engine uses frequency and statistical engines. The statical correlator, which correlates the image in the spatial and frequency domains, uses the Fourier domain, which identifies subpixel surface changes in phase images. However, the frequency correlator gives better results in terms of spatial correlation^77^. In order to identify surface deformation, the approach simultaneously employs a reference image from a prior period and a target image from a later time, both of which are changed to the frequency domain. Ground deformation in the target image is calculated with the help of the reference image^48^.

This study demonstrates multi-temporal optical imagery for monitoring deformation in the Joshimath area using COSI-Corr method. The land deformation activities are frequent in mountainous regions and pose a significant threat to populations^52^. In the context of land deformation, the COSI-Corr method can be used to identify the area where the ground subsidence movement is present and also track the ground displacement in the area over the time^54^. This displacement can be helpful to understand the area of land deformation and its causes by integrating it with other contributing factors^77^.

## Results and analysis

### Land Deformation estimation using SAR datasets for various periods in Joshimath

The Line of sight (LOS) land deformation velocity for the Joshimath region was estimated using PSInSAR based approach for the various periods with VV polarization channel of Sentinel-1 InSAR datasets. Figure 5a shows the land deformation velocity estimated during 2016–2017 using interferometric stack consisting of 12 SAR datasets. The obtained range of deformation for this period was − 40.57 mm/year to + 60.21 mm/year. The land deformation during period of 2018–2019 was estimated using interferometric stack consisting of 24 SAR datasets. The obtained rate of land deformation during this period was in the range of − 41.62 mm/year to + 12.39 mm/year. Further, the land deformation for 2020–2021 was estimated to in the range of − 39.767 mm/year to + 21.099 mm/year using 16 datasets Here, + ve sign signifies the LOS velocity/displacement away from the SAR sensor and − ve sign signifies the earth movement towards the SAR sensor in the direction of LOS. The region has experienced this rate of moderate land deformation during this periods 2022–2023.

**Figure 6:**
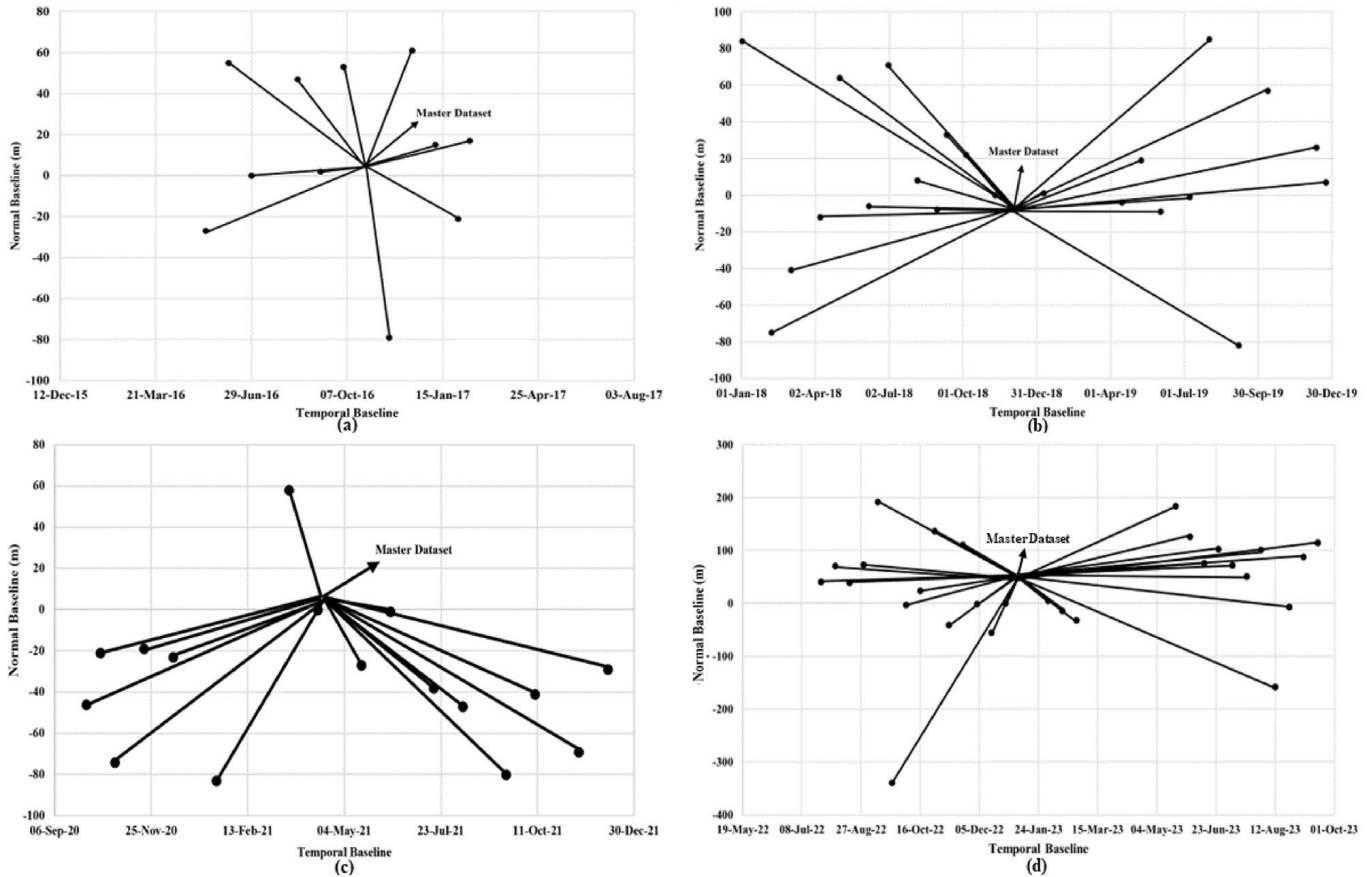
Temporal and Spatial baseline details for the SAR, (**a**) 2016–2017, (**b**) 2018–2019, (**c**) 2020–2021, (**d**) 2022–2023 (This figure is generated using Matlab 2023b https://in.mathworks.com/products/new_products/latest_features.html).

During the year 2022–2023, a sudden increase in the land deformation has been observed in the region as shown by the Fig. 5d. The Figure shows the estimated land deformation velocity in mm/year using 29 SAR datasets on google earth image background. The estimated LOS deformation velocity range was in the − 89.326 mm/year to + 94.46 mm/year range. As seen in Fig. 5d, maximum land subsidence is observed in the north-western region of the town, the Zone-1. The observed subsidence in Zone-1 is in the range + 60.45 mm/year to + 94.46 mm/year. Areas around Zone-2 in the north and central region of the town has also experienced high to moderate subsidence + 10.45 mm/year to + 60.45 mm/year. By contrast, an expansion is observed in the south-west region shown as Zone-3. The deformation velocity in Zone-3 ranges from − 89.65 mm/year to − 13.13 mm/year. Probable reasons for this land expansion can be water accumulation from the drainage system in the town below the rock debris and due to deposition of silt and continuous landslide activities in the region. Towards south-east of the town, the Zone-4 also experienced a rapid land subsidence − 13.13 mm/year to − 5 mm/year. The maps in Fig. 5 were generated using ArcGIS software-version 10.3.1. The spatial and temporal baseline information of the datasets used here in this study has been shown here in Fig. 6. The UAV acquired site photographs of the developed cracks due to land deformation events are shown in the further section.

Future SAR satellite missions, including NISAR (NASA-ISRO Synthetic Aperture Radar), RCM (RADARSAT Constellation Mission), ALOS-4, BIOMASS missions, and Tandem-L, will be providing extensive time-series interferometric datasets, which can be utilized comprehensively to investigate the causes and consequences of land surface alterations worldwide. These missions will also be providing derived products such as soil moisture and interferometric coherence, which can also be utilized in the applications of landslide mapping and monitoring^78,79^. NISAR will be one of the most important and crucial future SAR missions, which is being developed in the join collaboration of NASA and ISRO both which is scheduled to launch in year 2024. The NISAR mission will be NASA's first L-band SAR mission in orbit. It will be collecting high-resolution L-band SAR data (and S-band data in some regions) globally, it will be providing open access to the datasets which it will be acquiring every 12 days over the course of its 3-year mission^79,80^. This dual-frequency SAR satellite aims to comprehensively investigate the causes and consequences of land surface alterations worldwide, making it an invaluable tool for researching ecosystem disturbances, ice sheet dynamics, and natural hazard assessments^80,81^. NISAR is expected to greatly enhance our understanding of natural hazards and deepen our insights into the significant impacts of climate change^80,82^.

### Land deformation analysis using optical datasets

**Figure 7:**
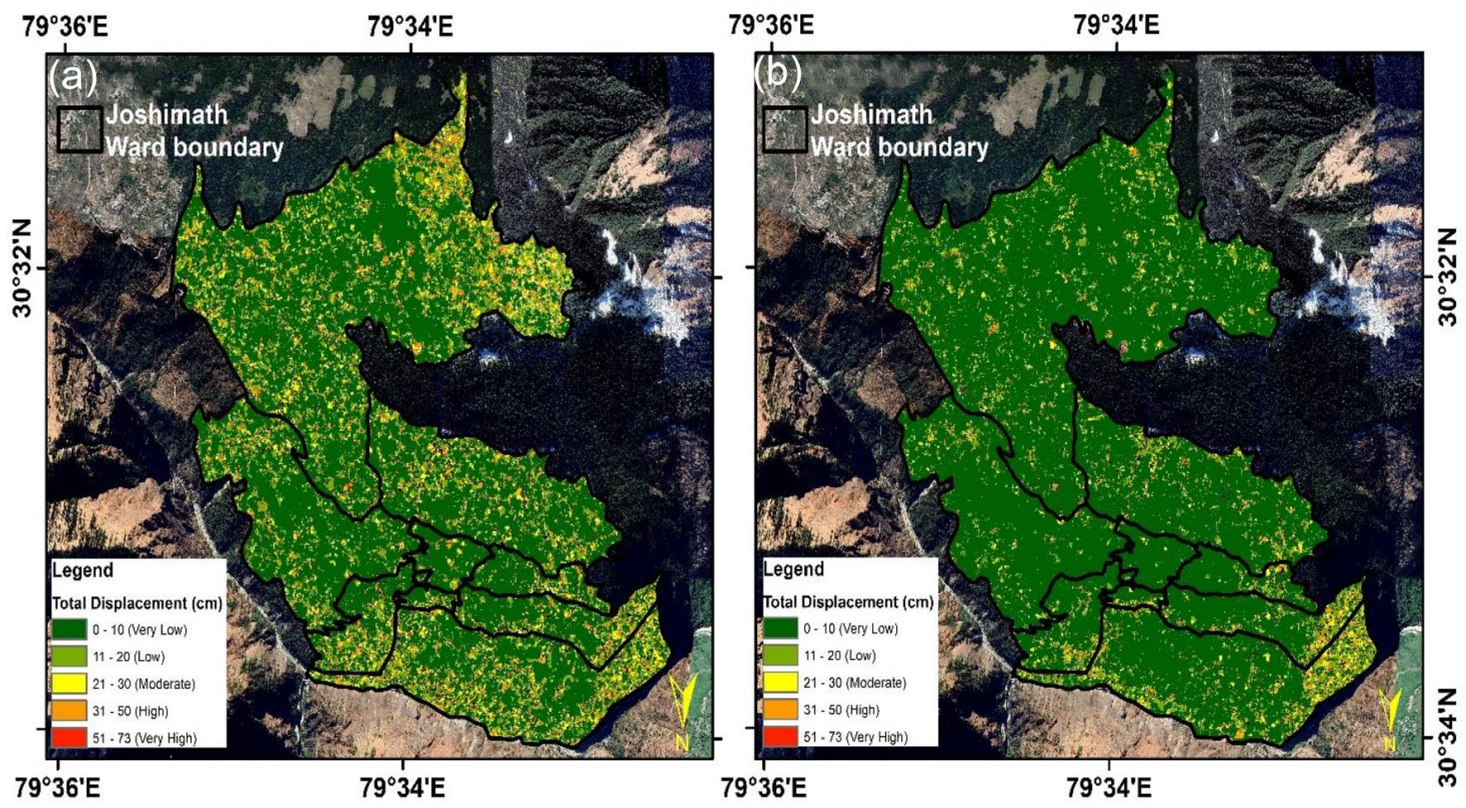
(**a**) Land deformation measurements using Cossi-Corr Methods. (**a**) December 04, 2020 to December 04 2021; (**b**) December 04, 2021 to December 28 2022 (This figure is generated using ArcGIS software-version 10.3.1https://enterprise.arcgis.com/en/portal/10.3/use/deploy-app-portal-obsolete.htm).

In this investigation, frequency correlator is employed utilizing Cosi-Corr techniques, where the X and Y domain windows are 32–64 with a 1-step size. It is generally accepted that larger windows produce a smoother effect, whereas smaller windows are more susceptible to background noise. The study used an image pair of Landsat 8 OLI sensors with a spatial resolution of 15 m, as well as Planet-Scope images with a 3-m resolution, to detect the land displacement. The images from December in the preceding year of 2022 minimize the uncertainty by having similar solar zenith angles. Table 2 displays the dates of the reference and target images for image pairings. Figure 7a and b shows the Land Deformation measurements using Cossi-Corr Methods from December 04, 2020 to December 04 2021 and December 04, 2021 to December 28 2022 generated using using ArcGIS software-version 10.3.1.

During the years 2021 and 2022 Joshimath showed some displacement in lower regions (Fig. 7b). December 27, 2022, to 10 January 2023 images reveal substantial displacement in the deformation region. In addition, the study found a lack of noticeable displacement in the years 2020 and 2021(Fig. 7a). By contrast, there was some displacement in Joshimath town's lower regions during 2022 and 2023. According to Cosi-Corr techniques, the displacement is between 40 and 55 cm, which is a decent sign of slow mass motions in the region.

**Figure 8:**
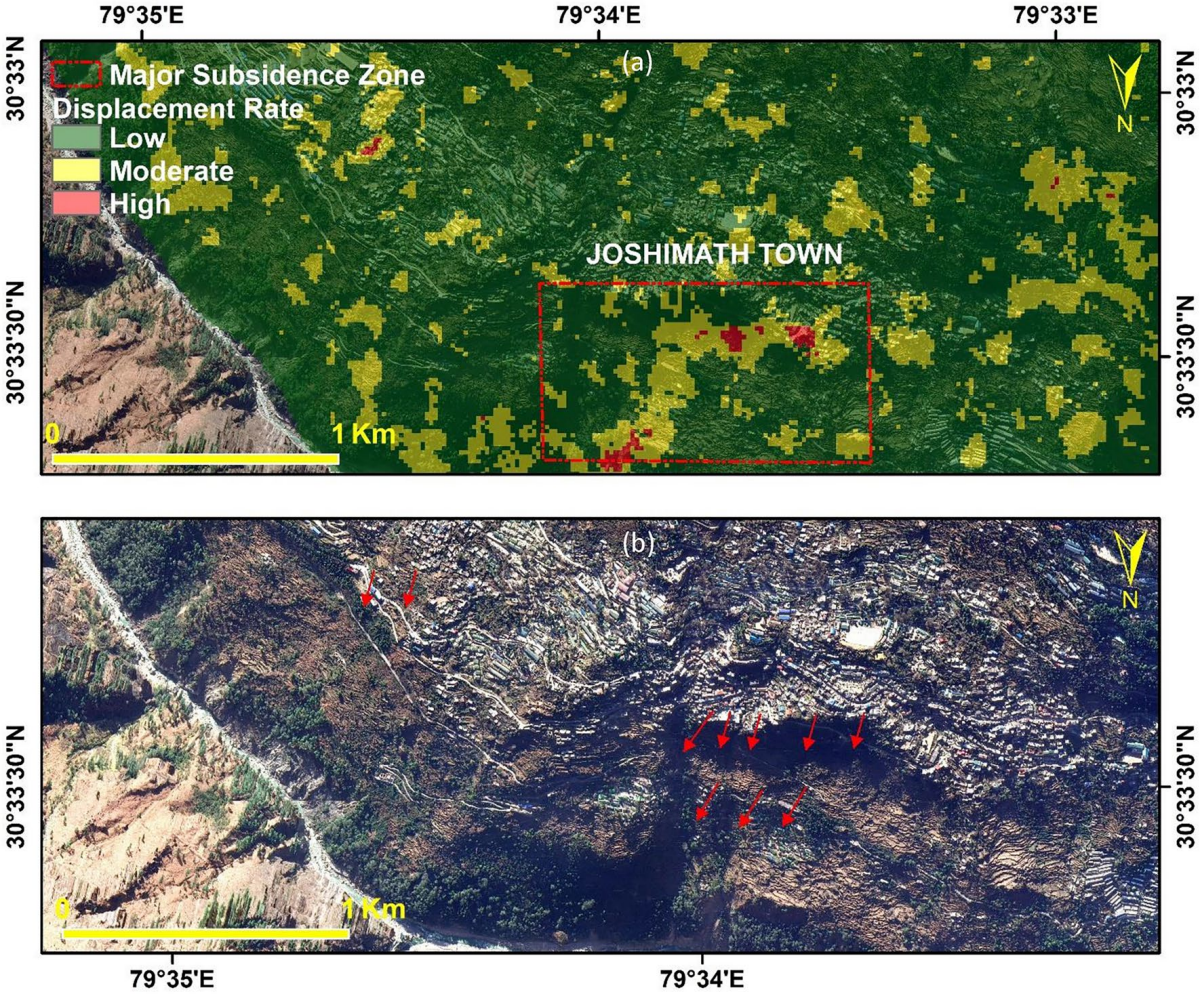
(**a**) Subsidence Risk Zones classification as High, Medium and Low based on the rate of displacement (**b**) Visual representation of the direction of subsidence measurements using Cossi-Corr Methods (This figure is generated using ArcGIS software-version 10.3.1https://enterprise.arcgis.com/en/portal/10.3/use/deploy-app-portal-obsolete.htm).

The slow-moving Identification of land deformation areas using optical imagery and Cossi-Corr methods is used for further zone identification. The area is classified into low- to high level classes based on the displacement rate. Figure 8 shows the subsidence risk zones classification as High, Medium, and Low depending upon the rate of displacement overlaid on Google Earth imagery for the visual representation of the direction of subsidence measurements using Cossi-Corr Method.

The arrows in Fig. 8b represent the movement of area from the field and drone datasets. The high movement or subsidence zone is identified by optical image correlation techniques. The High to low subsidence zone pattern shown in Fig. 8a) was recognized for mitigation and management purposes. The High zone mainly accumulates in an open slope area, which is also prone to land deformation in the future.

### Rapid urbanization in the region

**Figure 9:**
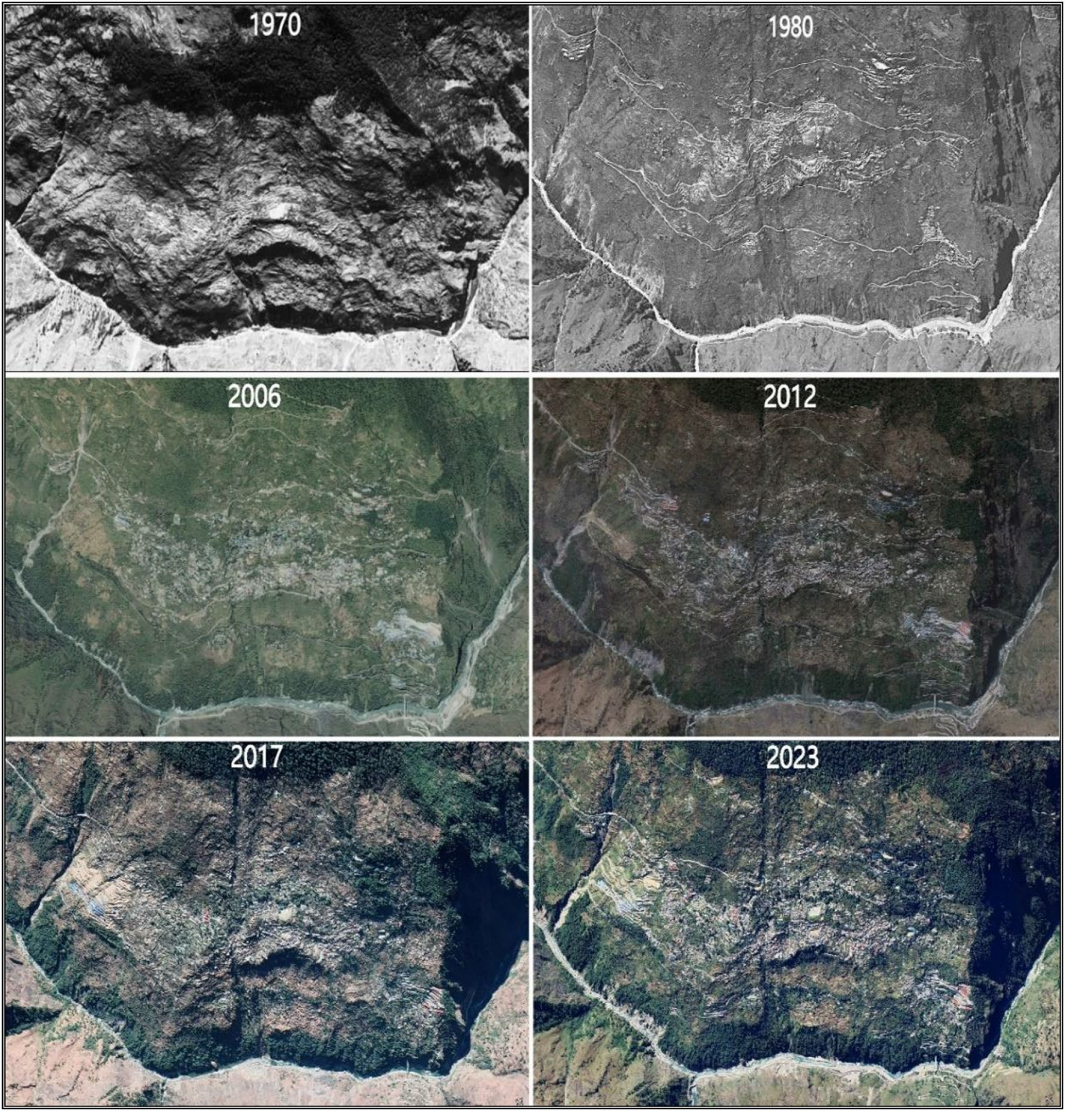
Rapid urbanization and building constructions in Joshimath. (*Source*: Google Earth and Corona datasets & Figure is generated using ArcGIS software-version 10.3.1https://enterprise.arcgis.com/en/portal/10.3/use/deploy-app-portal-obsolete.htm).

The sinking town of Joshimath has undergone a significant surge in built-up extent during the last few decades (Fig. 9). The changes due to rapid urbanization and building constructions in Joshimath in the years 1970, 1980, 2006, 2012, 2017, and 2023 are deciphered using the Corona datasets along with Google Earth datasets. The town has also witnessed a rapid increase in the annual number of visiting tourists. This has resulted in the establishment of many hotels and guest homes with multiple stories throughout the area. Also, the employment prospects for the population, particularly in the tourist and hotel industries, have led to a significant migration from the higher altitude and remote portions of the district to the town of Joshimath. Population growth has led to the development of commercial buildings and residences, roads, and transit systems for the local populace, placing heavy stress on the subsoil. The most recent census survey for Joshimath town was conducted in 2011, revealing that the town is divided into nine wards. According to the survey, the total population of Joshimath Nagar Palika Parishad was 16,709, with 9988 males and 6721 females, as reported by Census India in 2011. However, recent census data is not available. The Census survey, scheduled for 2021, has not been done until now.

**Figure 10:**
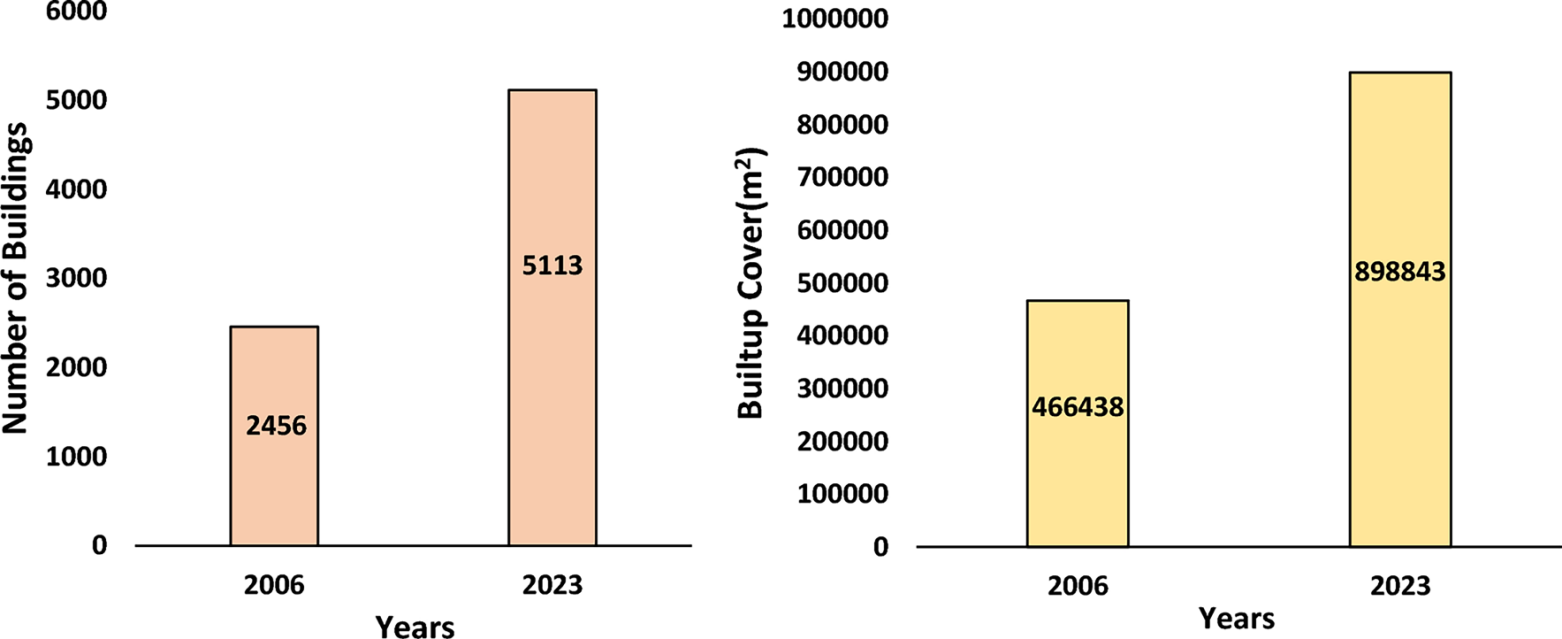
Statistics of the constructed houses and built-up in Joshimath in the years 2006 and 2023 respectively.

**Figure 11:**
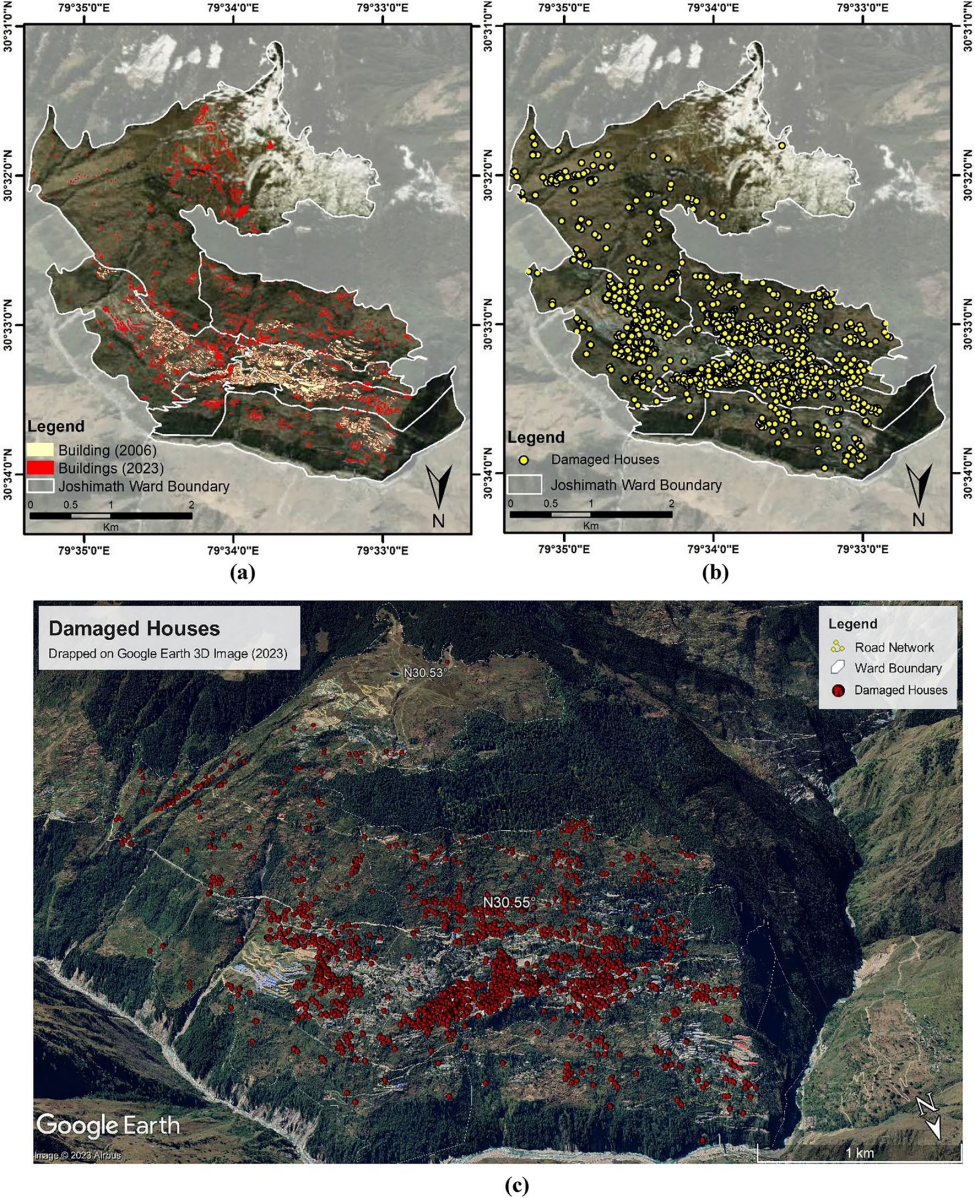
(**a**) The built-up covers in 2006 and 2023 respectively in the Joshimath Ward Boundary, (**b**) Ward-wise geolocation of the damaged houses in Joshimath, (**c**) damaged building infrastructure shown on google earth image. (This figure is generated using ArcGIS software-version 10.3.1 https://enterprise.arcgis.com/en/portal/10.3/use/deploy-app-portal-obsolete.htm).

In 2006, the number of buildings in Joshimath town was 2456, covering a corresponding area of 4,66,438 sq. meters, and in 2023, the number of buildings in Joshimath town increased to 5113, covering a corresponding area of 8,98,843 sq. meters (Fig. 10). Figure 11a shows the build-up cover area in 2006 and 2023, respectively, in the Joshimath Ward Boundary. This build-up cover area was extracted using the multi-temporal high-resolution Google Earth images for the years 2006 and 2023. The built-up areas in both years were manually extracted and marked separately. This approach allowed for the comparison and analysis of changes in built-up areas between 2006 and 2023 (shown in Fig. 11a). Figure 11b shows the ward-wise geolocation of the damaged houses in Joshimath based on the data from Uttarakhand State Disaster Management Authority, and Fig. 11c provides the damaged building infrastructure geolocations shown on Google Earth image. The map is genrated using ArcGIS software-version 10.3.1. The problem of cracks is prevalent among the majority of recently built infrastructures. The unplanned and improper construction activities in Joshimath town have flouted the construction norms, aggravating the region's vulnerability to hazards. Construction in a mountainous region can be challenging due to the rugged terrain and hazard risk, which emphasizes the need to consider factors such as slope stability, erosion, and the impact of natural disasters such as earthquakes and landslides before planning permanent structures.

**Figure 12:**
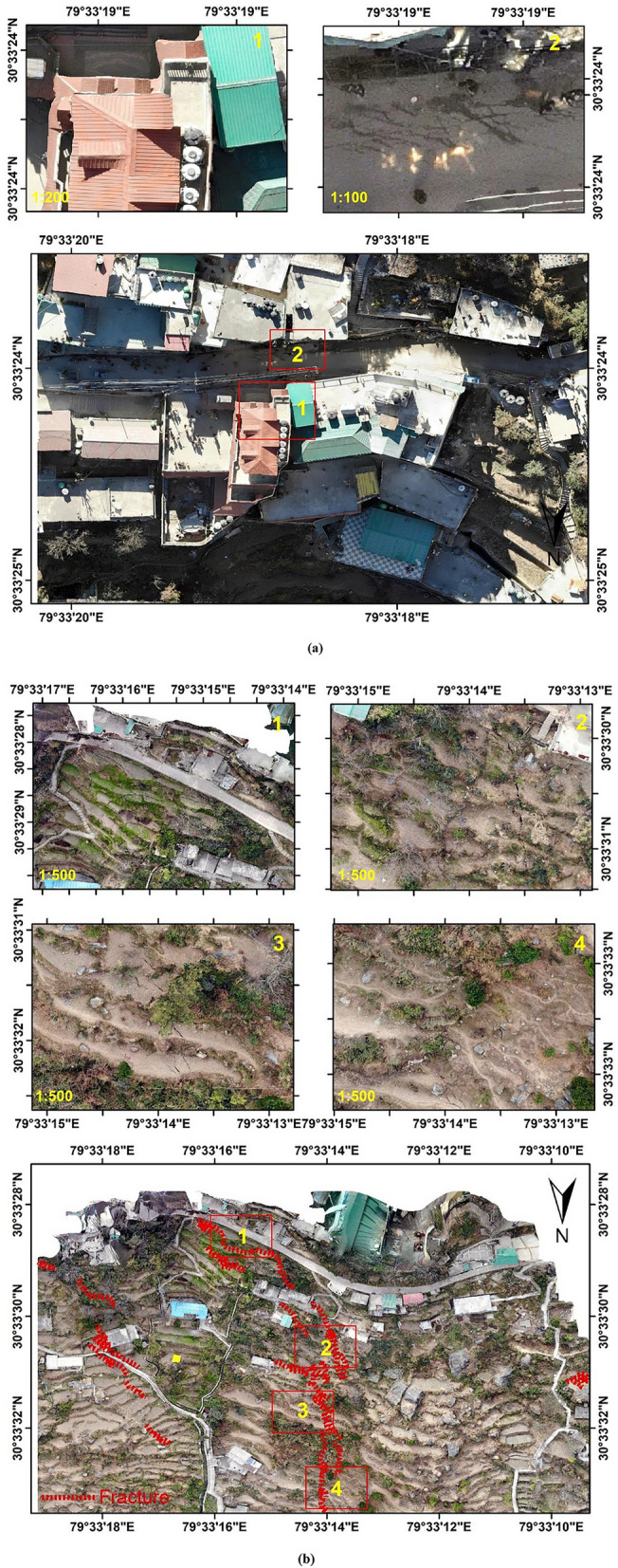

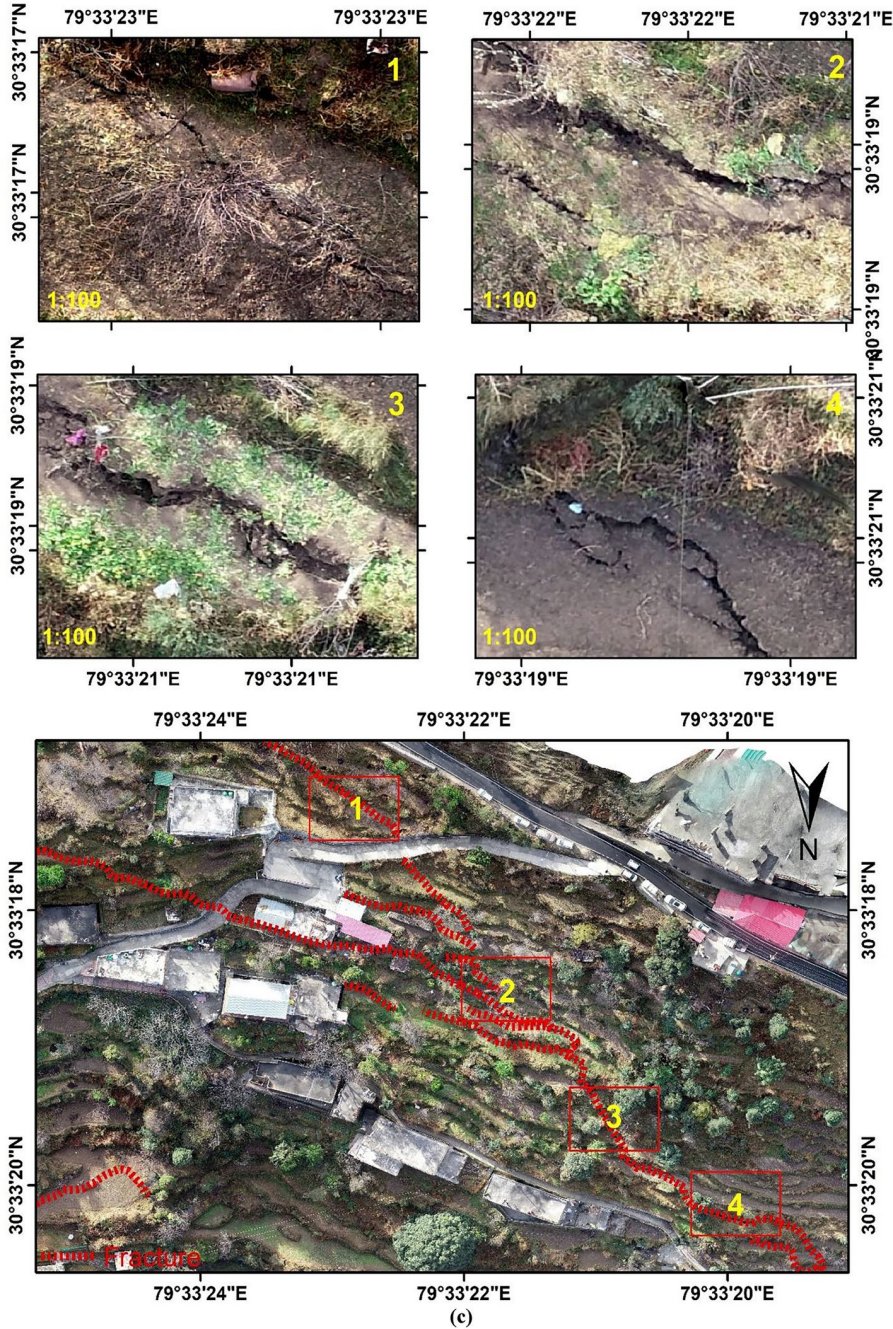
UAV acquired ground datasets from crack affected regions in Joshimath: (**a**) Hotel Mountain View and Malhari Hotel, (**b**) Singhdhaar region, (**c**) Manoharbagh region (This figure is generated using ArcGIS software-version 10.3.1https://enterprise.arcgis.com/en/portal/10.3/use/deploy-app-portal-obsolete.htm).

Figure 12a–c shows the ground images acquired using Unmanned Aerial Vehicle (UAV) of the crack-affected regions in Joshimath pre-processed using Pix4D software, and these maps were generated using the ArcGIS software-version 10.3.1. Figure 12a shows the UAV acquired datasets of the site of Hotel Mountain View and Malhari Hotel in Joshimath. It was observed that due to the sinking of the foundation of Hotel Malhari, the building tilted over Hotel Mountain view, making both buildings vulnerable to collapse. The cracks observed in the region of Singhdhaar area are shown in Fig. 12b using the UAV images of the site. At last, the UAV ground datasets were acquired for the Manoharbagh region, which also experienced a large number of cracks due to ground deformation and sinking activities as shown in Fig. 12c.

**Figure 13:**
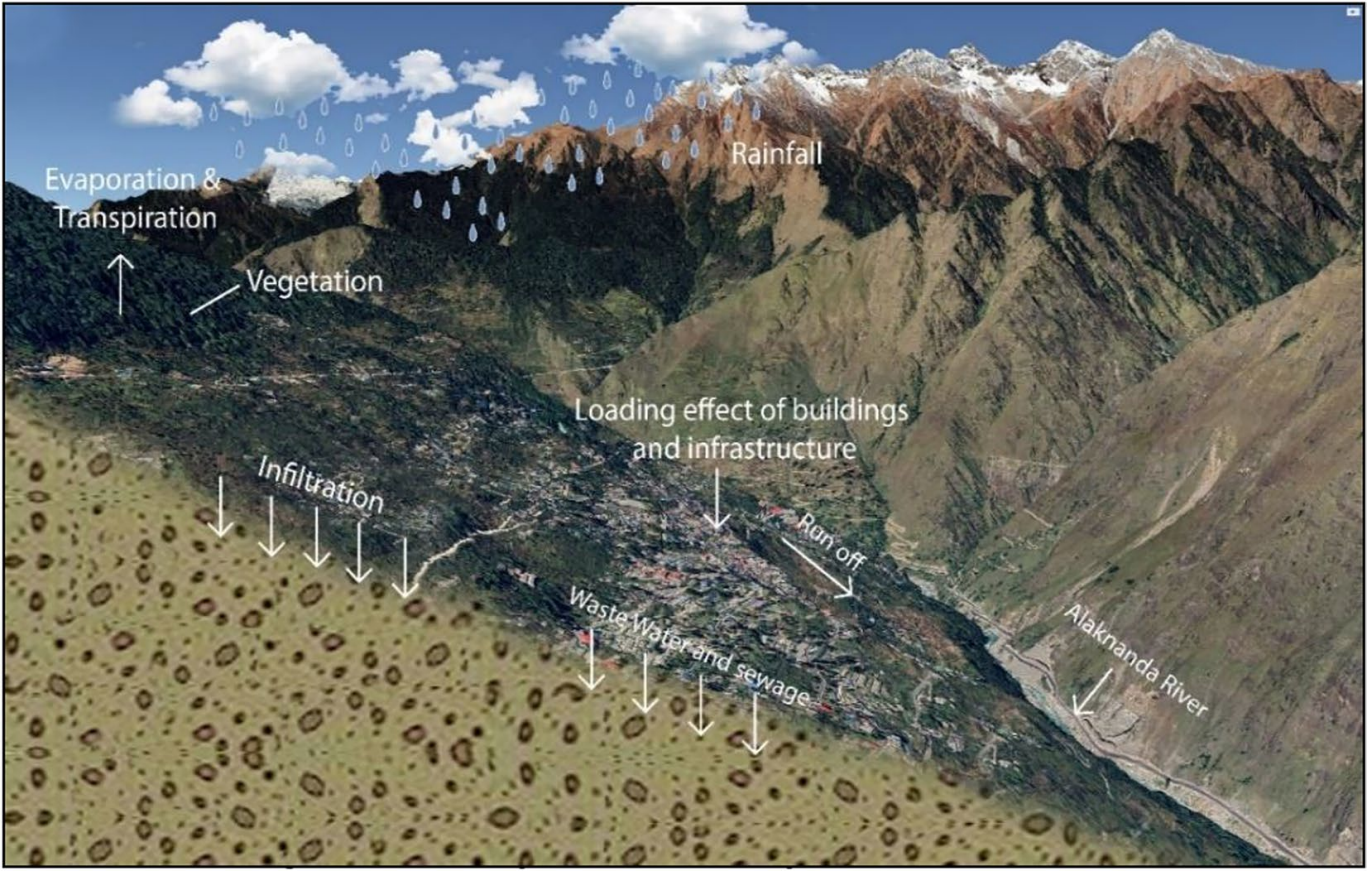
Schematic representation of water percolation in Joshimath town (This figure is generated using CorelDraw Graphics Suite 2019; https://www.coreldraw.com/).

**Figure 14:**
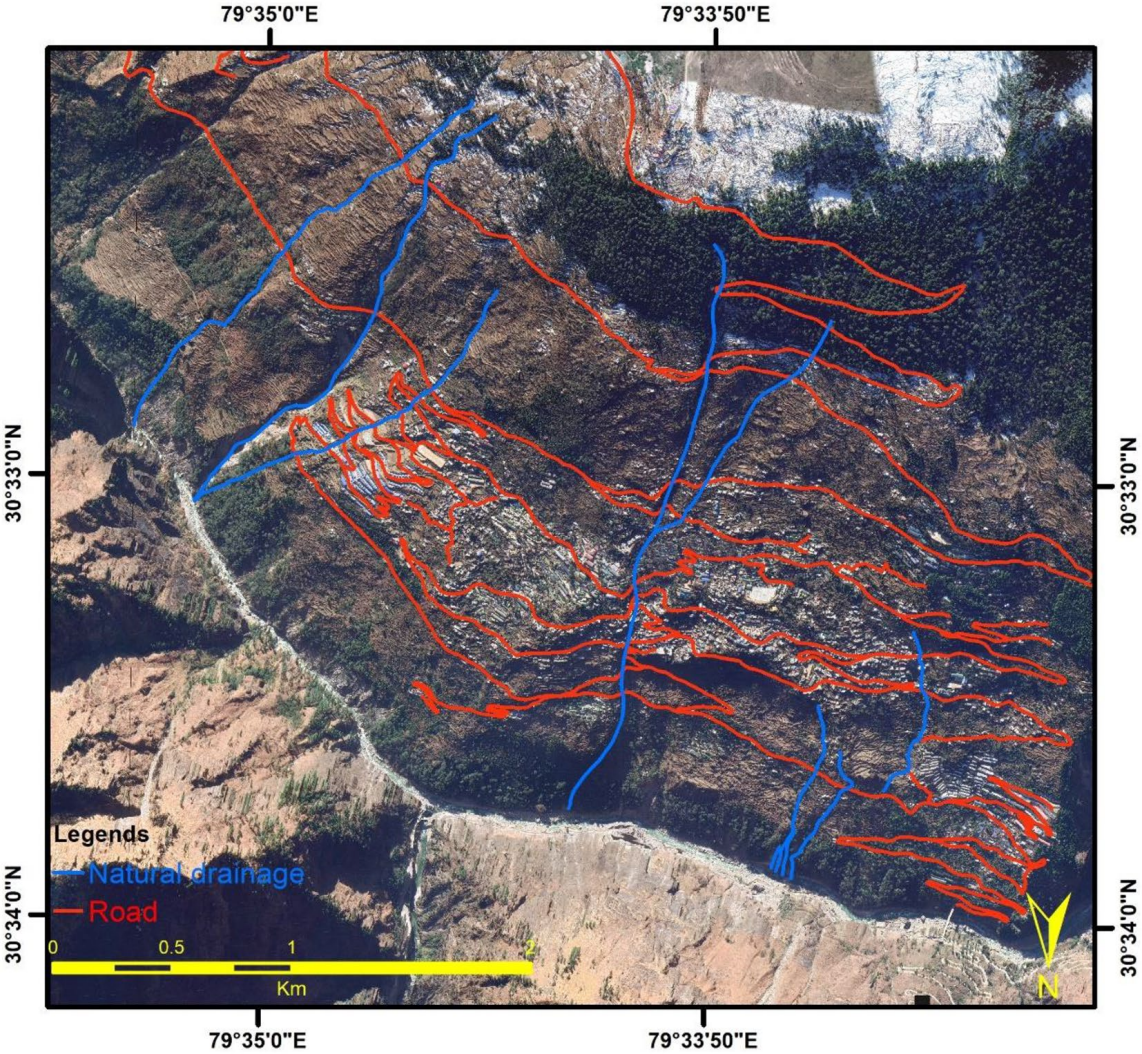
Natural Drainage system/Nala along with the road network in Joshimath town (This figure is generated using ArcGIS software-version 10.3.1 https://enterprise.arcgis.com/en/portal/10.3/use/deploy-app-portal-obsolete.htm).

Based on our ground data and field observations, it has been determined that the current drainage systems in the region are unplanned and inadequate. As a result, a significant portion of the rainfall is either infiltrating into the soil or running off through the sloping terrain without being properly managed or directed. Figure 13 shows the schematic representation of water percolation in Joshimath town based on the ground information. The lack of proper drainage systems combined with building constructions on natural drainages (Nalas) has caused blockages in natural water channels and increased water percolation into the soil, resulting in soil compaction and subsidence. As stated in the report given by the Disaster Mitigation and Management Centre (DMMC), there were nine nalas (natural drainages) in Joshimath^83^ in the year 2006, but due to the recent built-up and construction activities, most of these natural drainages were blocked and only five drainages exist ^83^ at present (Fig. 14).

As the region's population and number of tourists have grown significantly, more hotels, homes, and other forms of infrastructure have developed in the past decade^84,85^. However, the region's drainage and sewage systems have not been simultaneously developed to keep pace with this infrastructural development (Fig. 14). The lack of proper drainage systems has resulted in excess surface flow and sewage water infiltrating into the ground through cracks. The high fluid pressure in cracks results in the propagation of cracks, and their eventual rupture aggravates the land subsidence.

For preventing this, it is essential to construct proper drainage channels, stop underground water seepage, and have a comprehensive waste management system in place. The sewage tanks and used water from houses must not be allowed to seep into the ground, and the soaking pits should be closed. Instead, sewage water should flow through a sewage line and be deposited into concrete safety tanks, which should be guarded against seepage and located away from landslide zones. New drains should be constructed for carrying waste and rainwater, and cracks should be filled to prevent water from infiltrating the mountain rocks and causing landslides. Therefore, a comprehensive drainage system is essential to prevent land subsidence in Joshimath, including proper infrastructure, slope design, regular maintenance, and effective waste management.

### High erosion activity and toe cutting due to Alaknanada river

**Figure 15:**
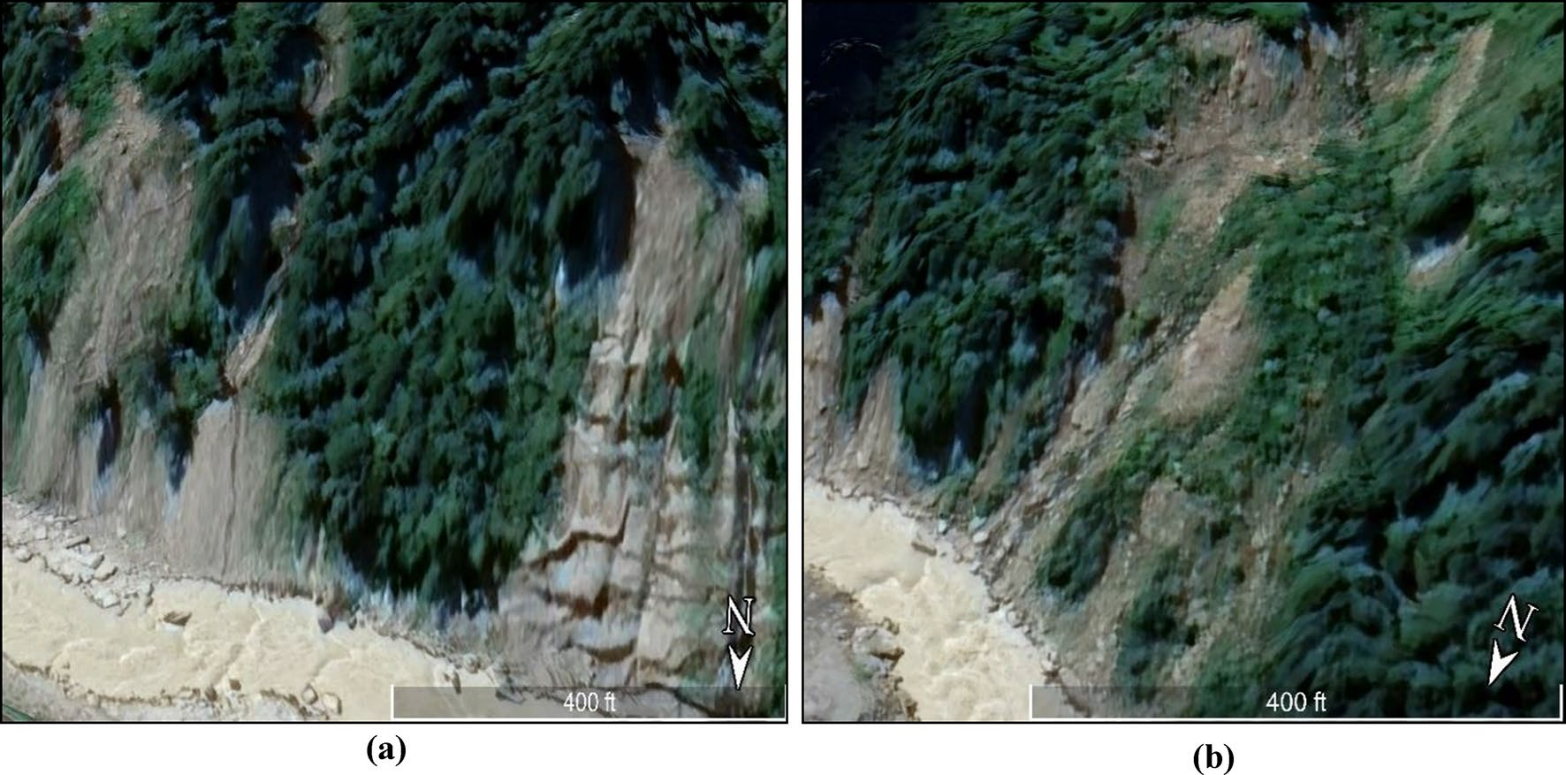
(**a**) and (**b**) Visualizations of Erosion and Toe Cutting due to Alaknanda River (This figure is generated using ArcGIS software-version 10.3.1 https://enterprise.arcgis.com/en/portal/10.3/use/deploy-app-portal-obsolete.htm).

In recent years, devastating flood events in the Joshimath region have caused significant erosion along the streams of the Alaknanda River, which flows from Vishnuprayag and passes through the town. The flash flooding in the Alaknanda and Dhauliganga channels exacerbated the situation by causing soil erosion beneath the town on the river's banks, rendering it more susceptible to instability. The remarkable flood events of June 2013 and February 2021 had a negative impact on soil stability, created landslide zones, and increased toe erosion and slope instability along Ravigram Nala and Nau Ganga Nala^68,70,86^. Also, during the flood event on 18 October 2021, the district of Chamoli received a very heavy rainfall of 81.9 cm for a duration of 4 h. Toe cutting, or the reduction of toe support, was observed in many areas along the Alaknanda and Dhauliganga rivers, particularly in the meanders of the rivers. Figure 15a and b visualizes the Erosion and Toe-cutting due to the Alaknanda River in the Joshimath region using Google Earth imageries.

Hard rocks are prevalent near the river bank in most areas, but loose soil and drifted boulders are present on the southern bank, indicating a zone of active river cutting^87,88^. The slow sinking of the town, along with the villages of Semkhurela, Sema, and Kamet, is a result of the scouring of the Alaknanda and Dhauliganga rivers, which carry away loose boulders and debris and cut into the hill slopes. To prevent further erosion, instability, and scouring of the hill toe on the slope south of Vishnuprayag and at the confluence of Alaknanda and Dhauli Ganga, retaining walls must be built for lateral support and prevention of toe cutting in affected areas. Furthermore, placing cement blocks on vulnerable parts of the river banks and wire crates filled with large boulders can also prevent scouring by the rivers and the slipping of loosely set terrace materials. In the long term, measures such as constructing stormwater drainage and sewage systems, retaining walls, and concrete blocks will need to be implemented to prevent further scouring by the rivers.

Erosion and its consequent sediment accumulation represent a subtle yet significant natural threat capable of disrupting the hydraulic dynamics within a river system^89^. In the Alaknanda basin, the rate of physical erosion is five times greater than the worldwide norm, and the Alaknanda River plays a crucial role in supplying sediments to the Ganga River^89^. Human activities such as the construction of dams and reservoirs are altering the basin's pristine landscape^90^. Therefore, it becomes imperative to give precedence to areas vulnerable to erosion, assess the degree of weathering, and pinpoint the specific bedrock sources responsible for the sediment influx^89^. The elevation profiles of rivers are frequently employed to interpret the tectonic and erosion history. As rivers flow downstream, the gradient of their channels typically decreases, a phenomenon that the river's concavity can describe. Further, a study by Flint^91^ formalized these findings into the slope–area relationship incorporating a concavity index (θ) to depict the rate at which river gradient decreases as drainage area increases and a steepness index (k_s_) that characterizes the steepness of a river reach independently of its drainage area.

According to the study by Flint^91^, the channel gradient decreases downstream systematically in a pattern that is explained by the ‘*Stream Power Model*’ written in the form of Eq. (5):

5 $$\frac{dz}{{dt}} = U - kA^{m} \left( {\frac{dz}{{dx}}} \right)^{n}$$

where A refers to upslope area, U is erosion uplift rate, $\frac{dz}{{dx}}$ is upslope, and $k,m$ and $n$ are coefficients.

In a steady state condition, $\frac{dz}{{dt}}$ = 0 at each point along the profile. Hence Eq. (6) is rewritten as:

6 $$\left( {\frac{dz}{{dx}}} \right)^{n} = \frac{U}{{kA^{m} }}$$

On further calculations it can be rewritten as Eqs. (6) and (7):

7 $$\frac{dz}{{dx}} = \left( \frac{U}{k} \right)^{1/n} A^{ - n/m}$$

8 $$S = k_{s } A^{ - \theta }$$

where S is $dz/dx$, concavity index (θ) is $m/n$, and $k_{s}$, the channel steepness index is equal to $\left( {U/k} \right)^{1/n}$, *θ* ranges between 0.3 and 0.8, and usually, the value is taken as 0.45°.

Many such studies show a clear positive correlation between k_sn_ and inferred erosion and uplift rates^25,92–94^.

**Figure 16:**
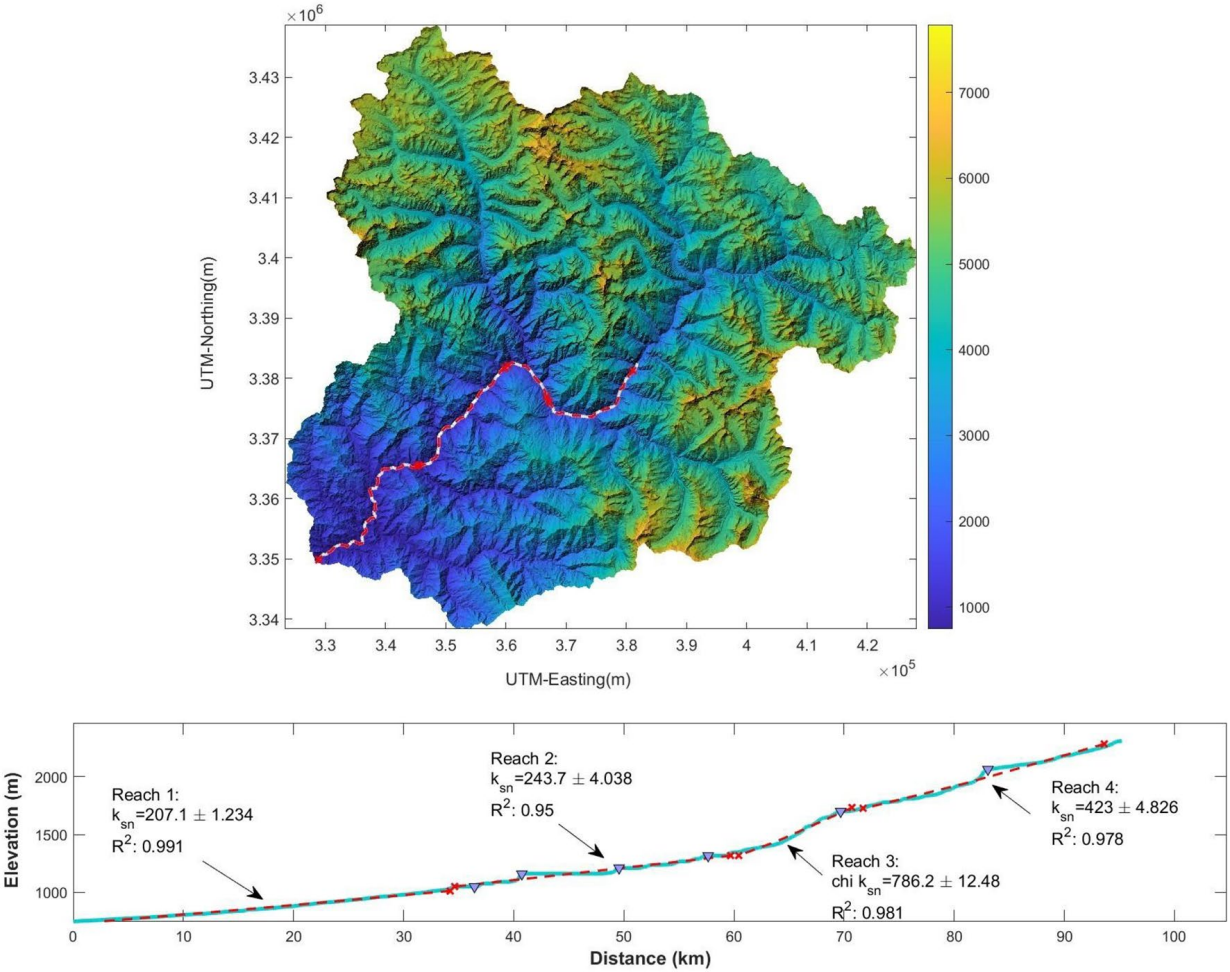
The longitudinal river profile along with the k_sn_.The red dashed line show the linear regression^95^ (This figure is generated using ArcGIS software-version 10.3.1 https://enterprise.arcgis.com/en/portal/10.3/use/deploy-app-portal-obsolete.htm and MATLAB R 2023b (Version 9.9 https://in.mathworks.com/products/mapping.html).

The linear regression analysis was performed through chi-elevation data, with the rivers being manually chosen for this process. Figure 16 illustrates how longitudinal river profiles are typically smooth, whereas the convexities (knick-points) in the stream datasets, which indicate erosional events and localized steepening of the stream, are revealed by discontinuities in the stream data sets. The middle reach of the basin exhibits a higher steepness value with a mean value of 786.2 ± 12.48, indicating a faster uplift rate and reflecting that the river is eroding faster around Joshimath. The high channel steepness correlates with the distribution of the knick-point positions along the tectonic fault line. The places near the Joshimath are situated in active faults, as shown in Fig. 16 which can also be activated because of unplanned construction and high urbanizations. The authors achieved comparable results within their respective study area.

**Figure 17:**
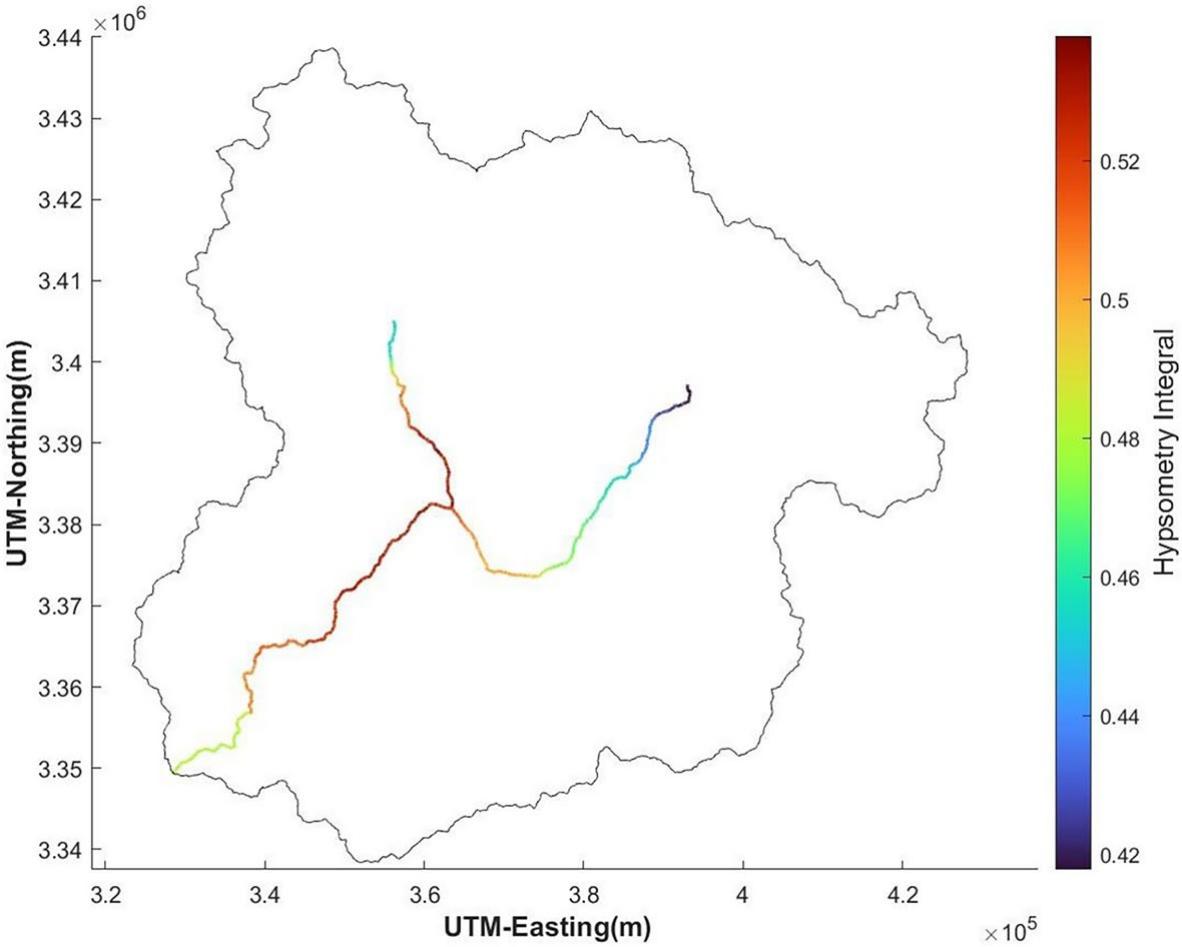
The spatial distribution of hypsometry integral values (This figure is generated using ArcGIS software-version 10.3.1 https://enterprise.arcgis.com/en/portal/10.3/use/deploy-app-portal-obsolete.htm).

**Figure 18:**
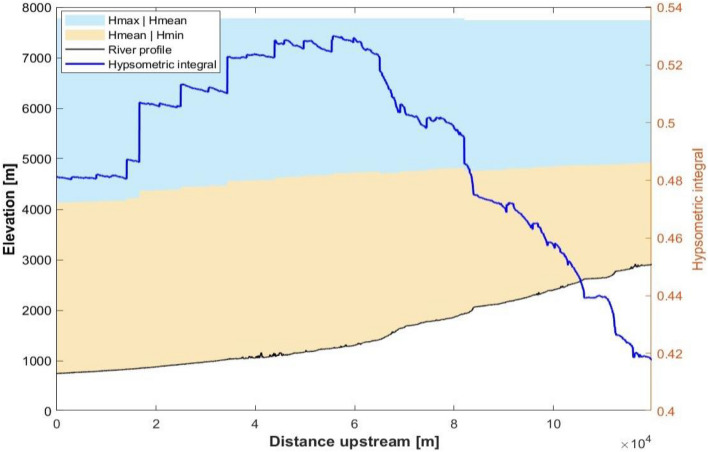
Downstream variation of hypsometry curve along the study area. (This figure is generated using ArcGIS software-version 10.3.1 https://enterprise.arcgis.com/en/portal/10.3/use/deploy-app-portal-obsolete.htm).

The hypsometry analysis aims to comprehend the erosional topography and tectonic activities of the area (Fig. 17). Further, the spatial distribution of hypsometry integral values has been shown (Fig. 18). The downstream variation in the Hypsometry integral along the catchment ranges from 0.42 to 0.5 as shown in Fig. 18. The Hypsometry Integral (HI) value is 48%, while the Erosional Integral (EI) is 52%, indicating a mature stage of a basin and subjected to toe erosion.

### Development of the huge infrastructure projects

**Figure 19:**
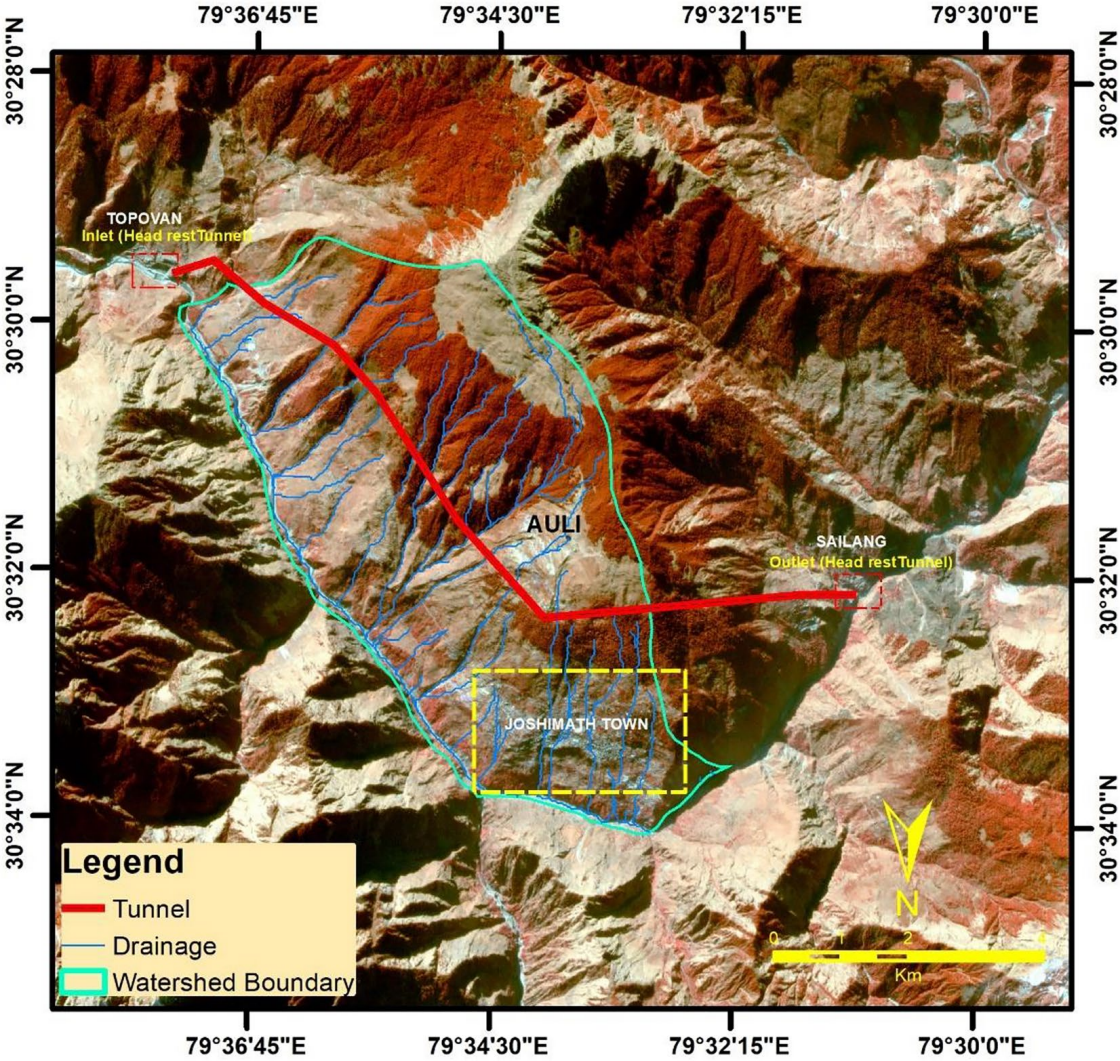
Hydropower Tunnel project shown in the Joshimath area along with the Watershed Boundary (This figure is generated using ArcGIS software-version 10.3.1 and CorelDraw Graphics Suite 2019 https://www.coreldraw.com/).

The development of infrastructure in a mountainous region, while necessary, is also one of the triggering factors for the increase in unstable or fragile region slopes. The region of Joshimath, located in the high-risk seismic zone (Zone V), is constantly threatened by seismic activities. The recent construction of large infrastructure and hydropower projects in the region may have accelerated the loss of land stability due to the blasting of rock foundations and the widening of roads. The hazard risks of construction in a mountainous region emphasize the importance of considering factors such as slope stability, erosion, and natural disasters such as earthquakes and landslides before building permanent structures. According to the Mishra Commission report, Joshimath is located on an old landslide zone and is sinking, leading to a recommendation to restrict heavy construction in the area^96^. However, despite the region being such geological and environmentally fragile, several infrastructural development projects have been established in the region. In December 2009, one such event resulted in the puncturing of the underground aquifer during the tunnel boring near Auli (Fig. 19). This resulted in a huge discharge of water from the aquifers^97^, which further led to the drying up of significant aquifers and springs in these region. Figure 19 shows the geolocation of the Hydropower Tunnel project in the Joshimath area along with the Watershed Boundary delineated using Cartosat DEM on planetscope imagery as background.

In the current scenario as well, the sudden outpouring of large amount of water can have numerous impacts, including the drying up of aquifers and springs, which would result in shortages of drinking water during the summer season. Reduced ground moisture and biomass availability could negatively impact the life support system of the masses, including floral and faunal diversity^97^. Before large projects such as hydropower projects, major infrastructure development, underground tunnels, or road widening are undertaken, a comprehensive assessment of the geological and geophysical impact must be conducted in and around the area^98^.

## Discussion

The ground deformation phenomenon has long been known in and around Joshimath, but it has become very rapid and alarming in recent years^85^. The town of Joshimath, located near the Munsiari Thrust and, the Main Central Thrust (MCT) at the contact of the Lesser and Higher Himalayas, experiences significant land subsidence and instability due to a combination of geological, geophysical and anthropogenic factors. Located in a seismically active zone, the Joshimath town is susceptible to natural disasters such as earthquakes, landslides, and ice rock avalanches.

Built on paleo-landslide debris, the town has exceeded the load-bearing capacity of the foundation. The major reason for this is the increased tourism and migration, resulting in the substantial growth of the hotels and tourist industries and the construction of multi-story hostels and guest houses, which have exceeded the load-bearing capacity of the debris underneath. Furthermore, the construction of five- or six-story hotel buildings, large infrastructural projects of highways, and enormous power projects are not sustainable for this region. All the construction work in the area should be minimized immediately to prevent further damage. All new construction should be constructed after a geotechnical investigation of the area. Building material must be lightweight and earthquake-resistant, which can help minimize the building load.

The Joshimath town lacks adequate sewage systems and drainage channels to sustain its rapid growth in recent years. It originally had nine natural drainages (nalas), but only five of them currently exist. This is because of the unplanned development that has changed the town's natural drainage pattern. Locals have also reported the subsurface seepage of water starting since 2014. In addition, the rapid construction of buildings has obstructed the city's natural drainage channels (Nalas). Inadequate drainage systems have caused excessive surface runoff and sewage water to infiltrate and seep into the ground, thereby accelerating land subsidence. Further, the flood events in June 2013 and February 2021 also negatively impacted this region. These events resulted in significant toe erosion along the streams of the Alaknanda River in the town's foothills and led to the formation of landslide zones that further increased toe erosion and slope instability.

To prevent land subsidence in Joshimath, a comprehensive drainage system is required, comprising proper infrastructure, slope design, routine maintenance, and efficient waste management. The septic tanks and household wastewater should not be permitted to seep into the ground, and the soaking pits should be sealed. Instead, sewage water should flow through a sewage line and be deposited in concrete safety tanks that are protected against seepage and located away from areas prone to landslides. To prevent water from infiltrating mountain rocks and causing landslides, drains should be constructed to transport water to safe areas, and cracks should be filled with a mixture of line, local soil, and sand bitumen. Further, in order to mitigate toe erosion and slope instability along the Alaknanda River, retaining walls must be constructed in affected areas to provide lateral support and prevent toe cutting.

Therefore, it is essential to acknowledge that the issues the town of Joshimath is facing are not unique to this region. Many other regions in the Himalayas and around the world face comparable environmental and geological challenges because of rapid human development. Therefore, a concerted effort is required to address these issues and promote sustainable development practices that place a premium on environmental preservation. Joshimath's future depends on the implementation of effective measures to mitigate environmental and geological hazards. Future imperatives include prioritizing sustainable development practices and implementing policies that account for the region's fragile geological and environmental conditions. The growth of the town should be regulated, and large infrastructure projects should be carefully planned and implemented. Large infrastructure and hydropower projects should be evaluated carefully to ensure that they do not exacerbate existing environmental issues. To prevent further land subsidence, the government should work towards developing an effective drainage system and waste management system while preserving the region's natural resources and taking into account the region's fragile geological and environmental conditions when planning and executing development projects. The vulnerability of the region to natural disasters such as earthquakes and landslides must be addressed, and precautions must be taken to protect the local population. In addition, the government should invest in the development of alternative industries, such as ecotourism and sustainable agriculture, to reduce the region's dependence on the hotel and tourism industries, which have contributed to land degradation. To ensure the long-term prosperity and well-being of the region, a cautious and sustainable approach is required overall.

## Conclusion

Our study provides the first quantitative estimates of the observed land subsidence in Joshimath town and an assessment of various causative factors, including geological, geophysical, and anthropogenic factors. The estimated land deformation velocity was in the range − 89.326 mm/year to + 94.46 mm/year. Uncontrolled population growth, unplanned built-up development, and inadequate drainage systems are the main causes that have intensified the situation. To mitigate the issue, the government needs to implement a comprehensive approach. This includes establishing a well-designed drainage system, implementing regular maintenance and effective waste management, and preventing water seepage and infiltration. Measures such as constructing retaining walls and employing erosion prevention techniques are essential to address toe erosion and slope instability. Immediate actions should involve minimizing construction activities, utilizing lightweight and earthquake-resistant building materials, and promoting sustainable development practices. The future of Joshimath relies on implementing effective strategies that consider the region's delicate geological and environmental conditions. This entails regulating urban growth, carefully planning and executing large-scale infrastructure projects, and diversifying industries. By doing so, the government can ensure the long-term prosperity and well-being of the region while preserving natural resources and safeguarding the population from natural disasters.

## Data Availability

The freely available raw datasets**-** Sentinel-1, Landsat-8, and declassified Corona datasets are available on ASF (https://asf.alaska.edu/) and Earth explorer (https://earthexplorer.usgs.gov/) and its processed and analyzed products are available upon request from the corresponding author. PlanetScope datasets, Drone Survey data and field-based damaged building survey data are provided to AG and GCJ for research purposes but are restricted by the government agencies from sharing.
