# Author Correction: Analyzing Joshimath’s sinking: causes, consequences, and future prospects with remote sensing techniques

**DOI:** 10.1038/s41598-024-68132-0

**Published:** 2024-08-09

**Authors:** Shubham Awasthi, Kamal Jain, Sashikanta Sahoo, Rohit Kumar, Ajanta Goswami, Girish Chandra Joshi, Anil V. Kulkarni, D. C. Srivastava

**Affiliations:** 1 Centre of Excellence in Disaster Mitigation and Management, Indian Institute of Technology Roorkee, Roorkee, Uttarakhand India; 2 Civil Engineering Department, Indian Institute of Technology Roorkee, Roorkee, Uttarakhand India; 3 Department of Earth Sciences, Indian Institute of Technology Roorkee, Roorkee, Uttarakhand India; 4 Uttarakhand State Disaster Management Authority, Dehradun, Uttarakhand India; 5 Divecha Centre for Climate Change, Indian Institute of Science, Bengaluru, Karnataka 560012 India

Correction to: *Scientific Reports* 10.1038/s41598-024-60276-3, published online 13 May 2024

The original version of this Article contained an error in Reference 61, which was incorrectly given as:

Dixit, R., Srivastava, D. C., Deshmukh, G. G. & Jain, A. K. *Main Central Thrust Zone (Mctz) and its Tectonic Boundaries, Alaknanda, Dhauliganga and Bhagirathi Valleys, Garhwal Himalaya, India. Alaknanda, Dhauliganga and Bhagirathi Valleys, Garhwal Himalaya, India* (2023).

The correct reference is listed below:

Mukherjee, P.K. et al. U–Pb zircon ages and Sm–Nd isotopic characteristics of the Lesser U–Pb zircon ages and Sm–Nd isotopic characteristics of the Lesser and Great Himalayan sequences, Uttarakhand Himalaya, and their regional tectonic implications. *Gondwana Res*. **75**, 282–297 (2019). 10.1016/j.gr.2019.06.001.

In addition, Figure 2 contained two red rectangles that were not representing the study area of the project. These have now been deleted.

Furthermore, the legend of Figure 2 contained an error.

“Geology map of the study region^62^ (This figure is generated using CorelDraw Graphics Suite 2019; https://www.coreldraw.com/).”

now reads:

“Geology map of the study region^61^ (This figure is generated using CorelDraw Graphics Suite 2019; https://www.coreldraw.com/).”

The original Figure 2 and accompanying legend appear below.

The original Article has been corrected.

**Figure 2:**
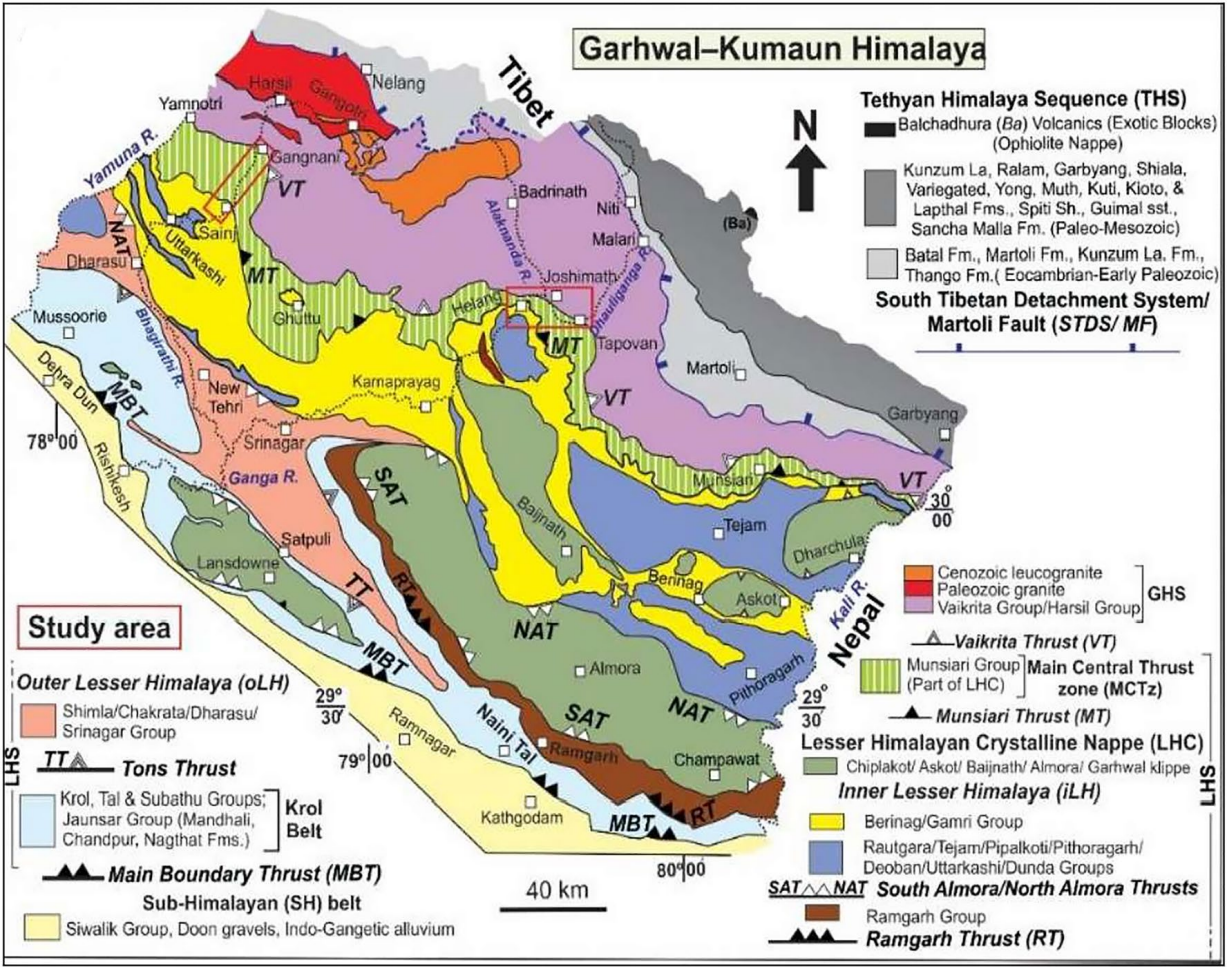
Geology map of the study region^62^ (This figure is generated using CorelDraw Graphics Suite 2019; https://www.coreldraw.com/).

